# Identification of *Padi2* as a novel angiogenesis-regulating gene by genome association studies in mice

**DOI:** 10.1371/journal.pgen.1006848

**Published:** 2017-06-15

**Authors:** Mehrdad Khajavi, Yi Zhou, Amy E. Birsner, Lauren Bazinet, Amanda Rosa Di Sant, Alex J. Schiffer, Michael S. Rogers, Subrahmanian Tarakkad Krishnaji, Bella Hu, Vy Nguyen, Leonard Zon, Robert J. D’Amato

**Affiliations:** 1 The Vascular Biology Program and Department of Surgery, Boston Children’s Hospital, Harvard Medical School, Boston, Massachusetts, United States of America; 2 Division of Hematology/Oncology, Boston Children's Hospital, Harvard Stem Cell Institute, Harvard Medical School, Boston, Massachusetts, United States of America; 3 Howard Hughes Medical Institute, Boston, Massachusetts, United States of America; 4 Department of Ophthalmology, Harvard Medical School, Boston, Massachusetts, United States of America; National Cancer Institute, UNITED STATES

## Abstract

Recent findings indicate that growth factor-driven angiogenesis is markedly influenced by genetic variation. This variation in angiogenic responsiveness may alter the susceptibility to a number of angiogenesis-dependent diseases. Here, we utilized the genetic diversity available in common inbred mouse strains to identify the loci and candidate genes responsible for differences in angiogenic response. The corneal micropocket neovascularization assay was performed on 42 different inbred mouse strains using basic fibroblast growth factor (bFGF) pellets. We performed a genome-wide association study utilizing efficient mixed-model association (EMMA) mapping using the induced vessel area from all strains. Our analysis yielded five loci with genome-wide significance on chromosomes 4, 8, 11, 15 and 16. We further refined the mapping on chromosome 4 within a haplotype block containing multiple candidate genes. These genes were evaluated by expression analysis in corneas of various inbred strains and *in vitro* functional assays in human microvascular endothelial cells (HMVECs). Of these, we found the expression of peptidyl arginine deiminase type II (*Padi2*), known to be involved in metabolic pathways, to have a strong correlation with a haplotype shared by multiple high angiogenic strains. In addition, inhibition of *Padi2* demonstrated a dosage-dependent effect in HMVECs. To investigate its role *in vivo*, we knocked down *Padi2* in transgenic *kdrl*:*zsGreen* zebrafish embryos using morpholinos. These embryos had disrupted vessel formation compared to control siblings. The impaired vascular pattern was partially rescued by human PADI2 mRNA, providing evidence for the specificity of the morphant phenotype. Taken together, our study is the first to indicate the potential role of *Padi2* as an angiogenesis-regulating gene. The characterization of Padi2 and other genes in associated pathways may provide new understanding of angiogenesis regulation and novel targets for diagnosis and treatment of a wide variety of angiogenesis-dependent diseases.

## Author summary

Angiogenesis plays a key role in a number of human diseases such as cancer, rheumatoid arthritis, cardiovascular disease, and macular degeneration. Evidence from our lab indicated that the ability to respond to angiogenic stimuli is controlled by genetic variation. This difference in angiogenic responsiveness could affect the susceptibility to a number of angiogenesis-dependent diseases. We have used the genetic diversity available in common inbred mouse strains to identify quantitative trait loci (QTLs) responsible for differences in angiogenic response and further refined the mapping of a genome-wide significant peak on chromosome 4. We then used both expression analyses and zebrafish model to successfully identify peptidyl arginine deiminase type II (*Padi2*), known to be involved in metabolic pathways, as the gene responsible for a significant portion of the difference in angiogenic responsiveness. In aggregate, these data are the first to indicate the potential role of *Padi2* as an angiogenesis-regulating gene and opens potential therapeutic avenues for a wide variety of systemic angiogenesis-dependent diseases.

## Introduction

Angiogenesis, the process by which new blood vessels are formed from existing vessels, plays a key role in a number of human diseases such as cancer, rheumatoid arthritis, cardiovascular disease, diabetic retinopathy and macular degeneration. Genetic variability in genes that control angiogenesis has been reported by several groups, and may influence susceptibility to and progression in angiogenesis-dependent diseases [1–3]. Regulation of angiogenesis is determined by a balance of pro- and anti- angiogenic signals and their interaction with endothelial cells and surrounding stroma. Important angiogenic regulators include vascular endothelial growth factor (VEGF), basic fibroblast growth factor (bFGF), angiopoietins (ANG1, ANG2) and platelet derived growth factor (PDGF). VEGF is normally released in response to tissue hypoxia, and acts on endothelial cells to increase cellular mitosis and migration [4]. VEGF activity is regulated by the expression of different isoforms and binding to different VEGF receptors and inhibitors [5]. bFGF is an extracellular matrix-bound protein normally released during wound healing. It signals through binding with fibroblast growth factor receptors (FGFR), a family of membrane receptors with tyrosine kinase activity [6]. Endogenous inhibitors of angiogenesis include soluble VEGF receptors, neuropilins, angiostatin, and thrombospondins, all of which are important in both health and disease.

Evidence from our lab and others indicates that the ability to respond to angiogenic stimuli is determined by genetic variation [7–12]. This difference in angiogenic responsiveness can affect susceptibility to a number of angiogenesis-dependent diseases. In addition, polymorphisms in *VEGF* have been associated with increased susceptibility to a number of cancers as well as eye diseases such as age-related macular degeneration [13, 14]. The identification and characterization of additional human genes contributing to angiogenic diseases will provide valuable new information regarding the underlying pathology of these diseases as well as suggest new methods of diagnosis and treatment. Genetic study of affected human populations is one path to identify novel genes. However, the successful identification of genes responsible for complex heterogeneous disorders such as macular degeneration requires a multipronged study design and very large, well-defined datasets of affected individuals [15].

In the case of studying complex traits, such as angiogenesis, a complementary approach is to use naturally occurring variants in experimental models such as common inbred mice. The availability of different strains of mice, produced through a variety of innovative molecular genetic techniques, affords the ability to identify and dissect traits through methodologies that cannot be used in humans. Furthermore, the ability to compare the complete genome sequence for the strains used in these studies greatly assists the rapid identification of biologically-relevant DNA sequence variants in potential candidate genes. The characterization of angiogenesis regulating genes in mice may help elucidate the molecular pathways responsible for modulating angiogenic responses in humans, thereby identifying excellent candidate genes for further investigations into genetic etiologies of pathological conditions. We previously observed significant differences in growth factor induced angiogenic responsiveness among different inbred mouse strains and demonstrated that this trait is genetically controlled by different loci [7]. We further used standard linkage based interval mapping techniques to identify a number of quantitative trait loci (QTLs) that contribute to differences in angiogenic response among select inbred mouse strains. In a few cases, we identified the specific genetic difference responsible for the QTL [12, 16]. Given the recent sequencing of many common inbred mouse strains [17], we used the genetic diversity available among these strains to perform a genome-wide association study to identify additional QTLs responsible for variation in angiogenic response.

The corneal micropocket neovascularization assay was performed on 42 different age-matched male inbred mouse strains using 20ng bFGF pellets. We next carried out high-resolution mapping in a large number of inbred mice using genome-wide association. To correct for population structure and genetic relatedness among inbred mouse strains, we employed Efficient Mixed-Model Association (EMMA) mapping and identified five QTLs with genome-wide significance on chromosomes 4, 8, 11, 15 and 16. We further refined the mapping in a genome-wide significant peak on chromosome 4, and used both expression analyses and zebrafish to successfully identify peptidyl arginine deiminase type II (*Padi2*), as the gene responsible for a portion of the difference in angiogenesis among strains. Padi2 is part of the PADI family of enzymes that is known to catalyze the peptidyl arginine residues to citrulline in the presence of Ca^2+^ [18]. This posttranslational modification could potentially affect the structure and ultimately the function of substrate proteins [18]. Padi2 is widely expressed in various tissues including central nervous system, retina, brain and skeletal muscle [19]. The characterization of Padi2 and other genes in associated pathways can open new avenues for its therapeutic use against a broad spectrum of angiogenesis-dependent diseases.

## Results

### Angiogenic response in common inbred mouse strains

**Fig 1:**
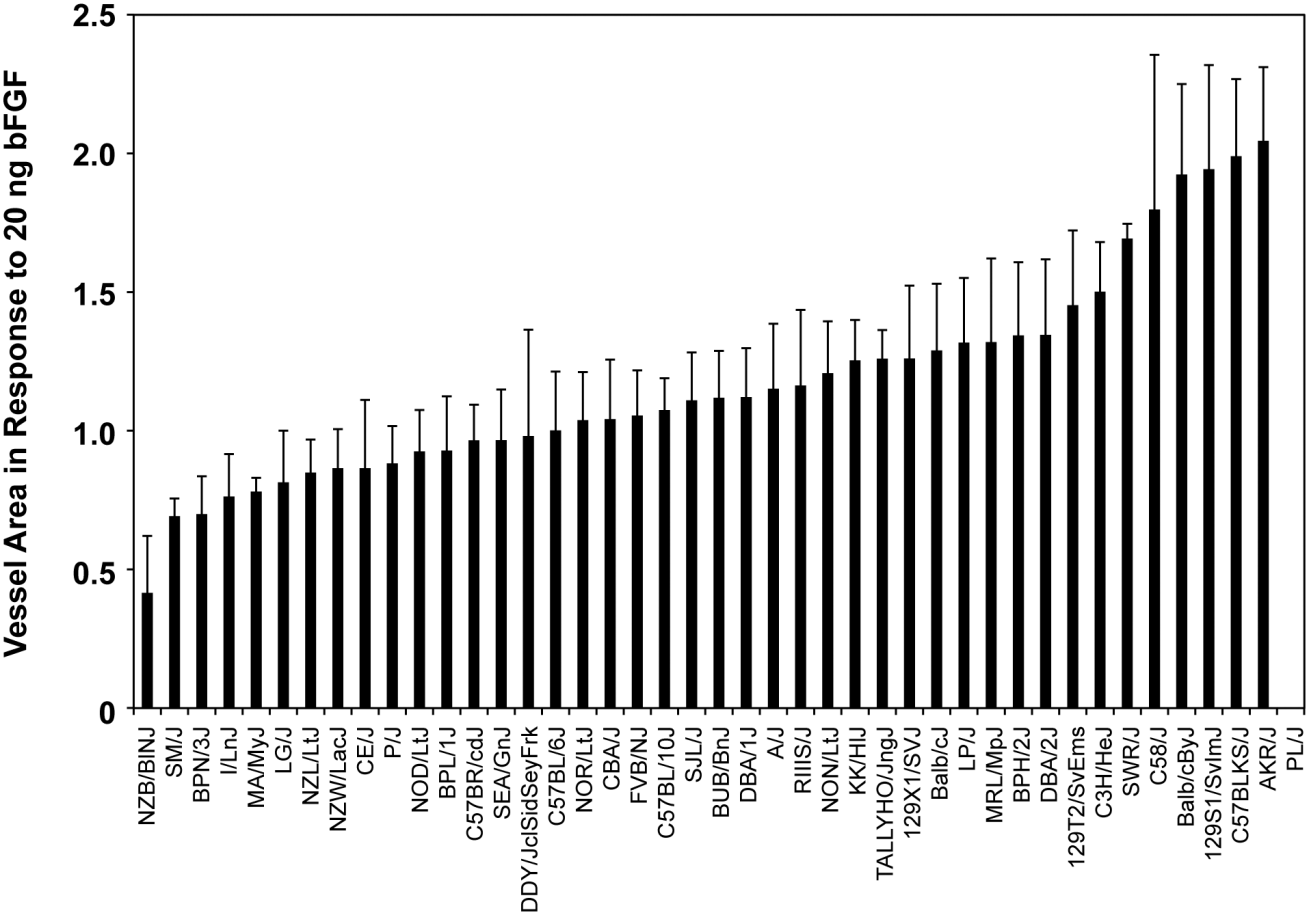
Angiogenic response in the cornea of common inbred mouse strains. Mean ± SD for measured vessel area using slow release 20 ng of bFGF pellets in cornea of 42 common inbred mouse strains. The difference between the strains with the lowest and the highest values were 4.88-fold. The cornea micropocket eye assay was not possible in PL/J mice due to proptosis.

In order to identify genomic regions associated with angiogenic response, we performed the corneal micropocket assay in 42 age-matched, male common inbred mouse strains. Briefly, 20 ng bFGF pellets are implanted in the mouse cornea 1.0 mm from the limbus. This dose of bFGF was designed to maximize the inter-strain variation in measured angiogenic response based on previously-reported responses [7]. Five mice (10 eyes) per strain were analyzed and similar numbers of control C57BL/6J were included in each assay to confirm consistency. We observed a broad and diverse range of measured vessel area across strains (S1 Note 1), from a value of 0.42 mm^2^ in NZB/BINJ (low angiogenic strain) to 2.05 mm^2^ in AKR/J (high angiogenic strain). Both are significant deviations from the mean value of the vessel area for all the tested strains (1.17 mm^2^) in this study (Fig 1). The broad distribution of data suggests the existence of multiple QTLs regulating responsiveness to bFGF.

### Genome-wide association to identify specific haplotypes with angiogenic response

**Fig 2:**
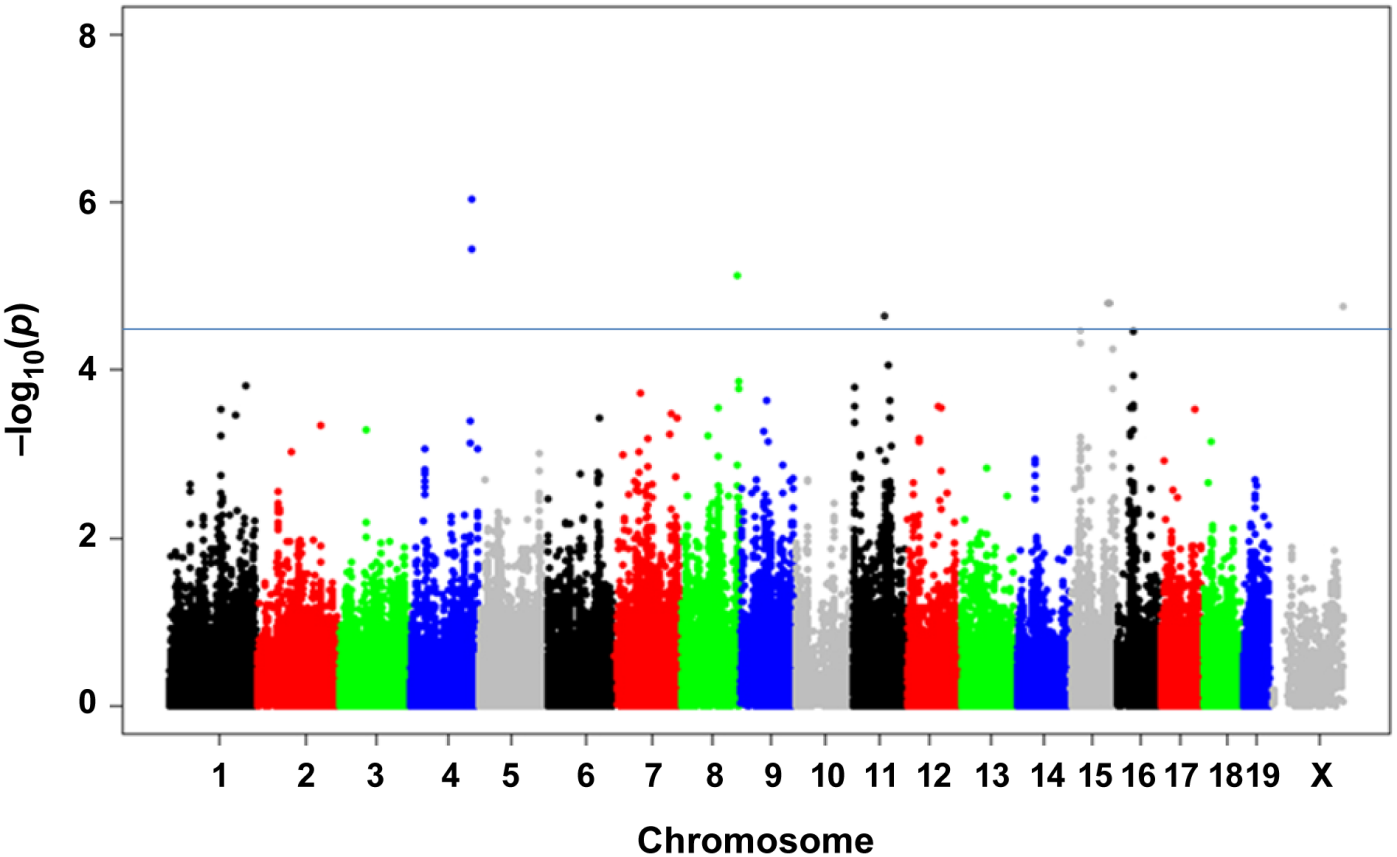
GWAS results for vessel area using EMMA. Manhattan plot showing the EMMA adjusted association (-log10) p-values (-logP) for vessel area in 42 common inbred mouse strains. The analysis was performed using 132k SNPs with a minor allele frequency > 5%. Each chromosome is plotted on the x-axis. SNPs on Chr. 4 (rs32857122; p = 9.34 x 10^−7^ and rs32259427; 3.67 x 10^−6^) show GWAS peaks with highest significance.

To determine which regions of the mouse genome are responsible for differences in angiogenic response, Efficient Mixed Model Association (EMMA) algorithm was applied [21] to account for interbreeding and to correct for population structure and genetic relatedness. Our analyses yielded five peaks with genome-wide significance (p< 0.05) on chromosomes (Chrs.) 4, 8, 11, 15 and 16 (Table 1, Fig 2). As expected, EMMA significantly improved the QQ-plot of the p-values (S1 Fig).

**Table 1:** Genome-wide association results of angiogenic response among common inbred strains.

Loci^1^	Chr.	Associated SNP	P-Value^2^	RefSeq Genes^3^
*AngRFq-1b*	4	rs32857122	9.34 x 10^−7^	3
*AngRFq-1c*	4	rs32259427	3.67 x 10^−6^	5
*AngRFq-3*	8	rs32840903	7.46 x 10^−6^	3
*AngRFq-4c*	15	rs33886061	4.75 x 10^−5^	8
*AngRFq-4d*	15	rs50093952	5.61 x 10^−5^	5
*AngRFq-6f*	16	rs52707645	3.46 x 10^−5^	10
*AngRFq-7c*	11	rs3688710	3.23 x 10^−5^	13

^**1**^ New angiogenesis QTLs are named *AngFq-* (for angiogenesis due to bFGF).

^**2**^ Genome-wide significant is set at p = 4.1 X 10^−5^ or -log10P = 4.39.

^**3**^ Number of RefSeq genes (NCBI´s Build37/mm9 assembly) located in the EMMA-linked region.

### Characterization of genome-wide significant peaks

**Fig 3:**
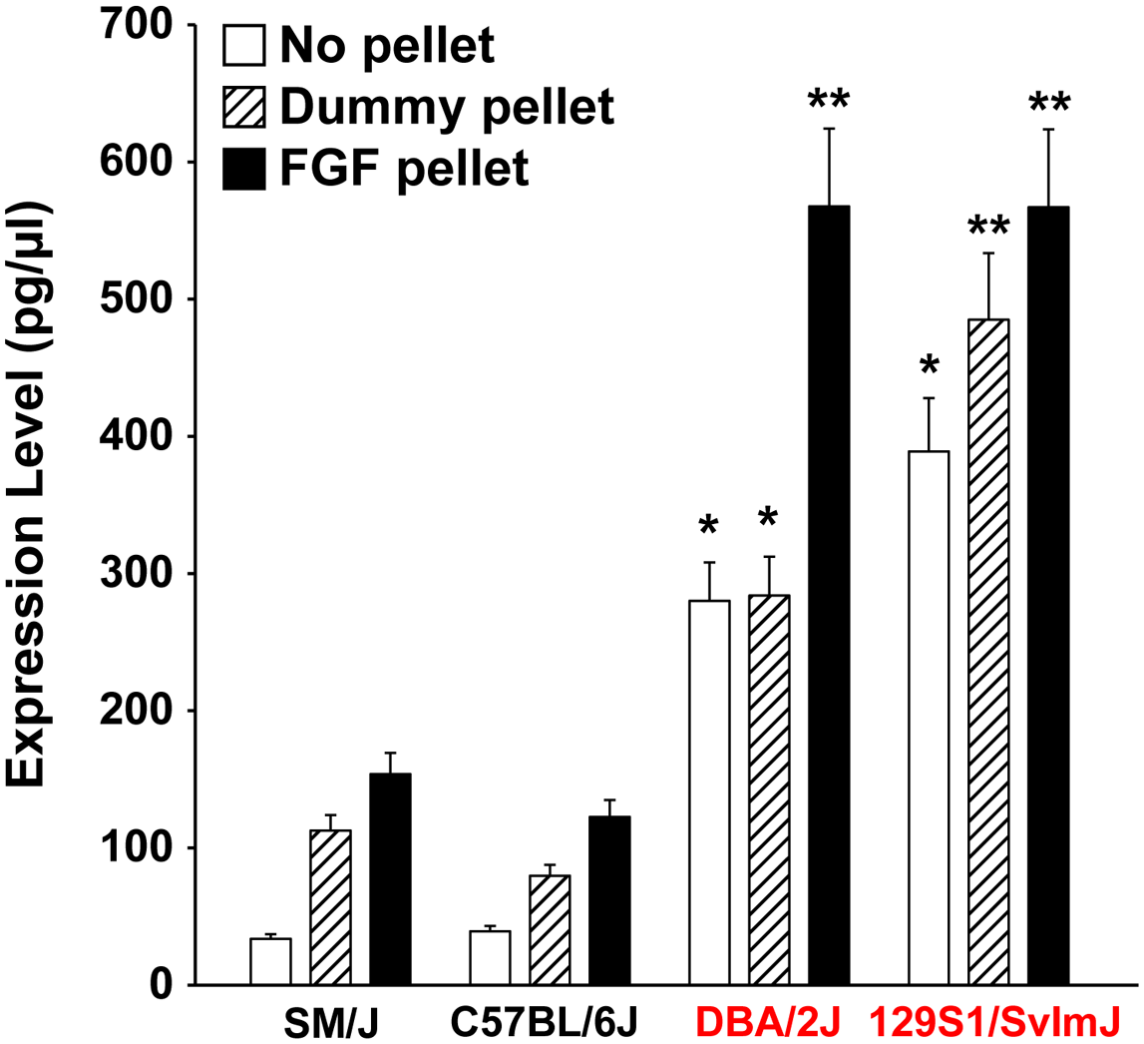
Gene expression of a candidate gene within the most statistically significant region identified by EMMA. Expression data of *Padi2* show a strong correlation with haplotypes among inbred mice who have “low” or “high” angiogenic responses. Coordinating colors represent inbred mice who share the same haplotype. Data represent mean ± standard error of the mean (SEM) from 8 age-matched corneas. * and ** indicates *P* < 0.01 and *P* < 0.001 respectively.

We next searched for a causal angiogenesis-regulating gene that was within haplotypes that are linked to differences in angiogenic responsiveness. Within each association peak there were 8 (Chr. 4), 3 (Chr. 8), 12 (Chr. 11), 13 (Chr. 15) and 10 (Chr. 16) unique reference sequences (Table 1). Gene selection criteria within the EMMA-linked regions were based on: candidate genes harboring a non-synonymous SNP within the coding region that may have functional consequences, or the presence of variants within the promoter or 5’-untranslated regions that may potentially alter gene expression. To identify genes with polymorphisms that affect gene mRNA levels in the relevant tissue, we next generated gene expression profiles using total RNA isolated from unstimulated corneas (no pellet), empty pellet with no growth factor (dummy pellet) and stimulated corneas with 20 ng bFgf pellet (8 eyes, excluding the limbus, pooled from 4 mice) of 4 different strains with varying angiogenic responses (SM/J, C57BL/6J, DBA/2J, 129S1/SvImJ). Subsequently, we examined the mRNA expression levels of each candidate gene by quantitative RT-PCR. On chromosome 4, expression data identified one candidate gene in the EMMA-linked region with a strong correlation of regions containing the same type of polymorphism among inbred mice who have “low” or “high” angiogenic responses suggesting the existence of an eQTL at this locus (Fig 3). We also identified another candidate gene, *Slc38A1* (Chr. 15) that did not show expression differences among inbred strains (S2 Fig) but bears a coding-nonsynonymous SNP (rs50093952) where the derived allele changes an amino-acid residue that is conserved in all mammalian species (S3 Fig). Notably, this SNP is only observed in a haplotype that is shared only among inbred strains with “high” angiogenic response.

### Deimination levels in the cornea

**Fig 4:**
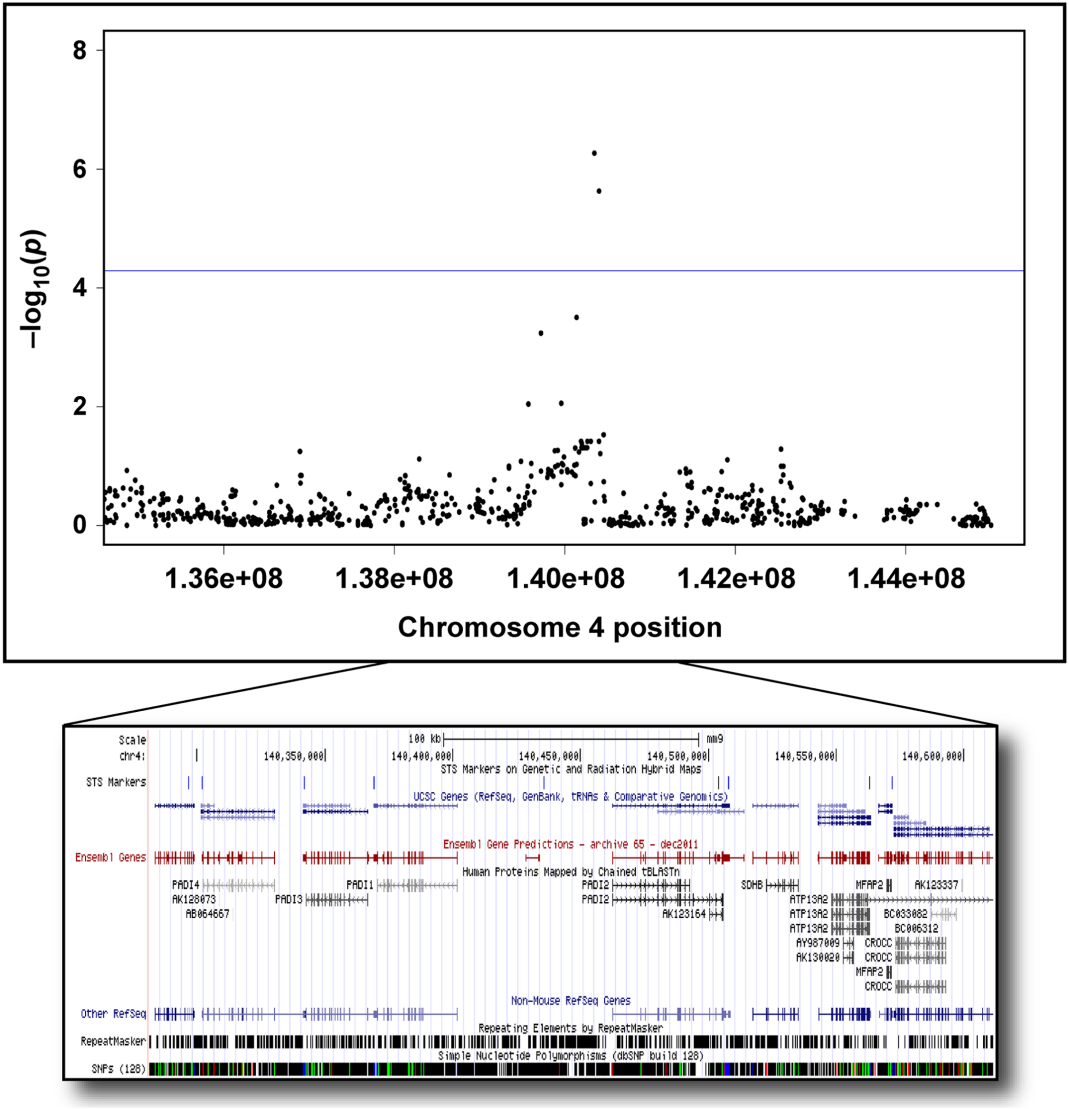
Regional plot of the Chr. 4 association in 42 common inbred mouse strains centered on the lead SNP (rs32857122) at the 5’-untranslated region of Padi2 locus. The top two black dots represents the most significant SNPs (p = 9.34 x 10^−7^ and 3.67 x 10^−6^ respectively). The positions of all reference sequence genes are plotted using genome locations from UCSC Genome Browser on Mouse July 2007 (NCBI37/mm9) Assembly.

**Fig 5:**
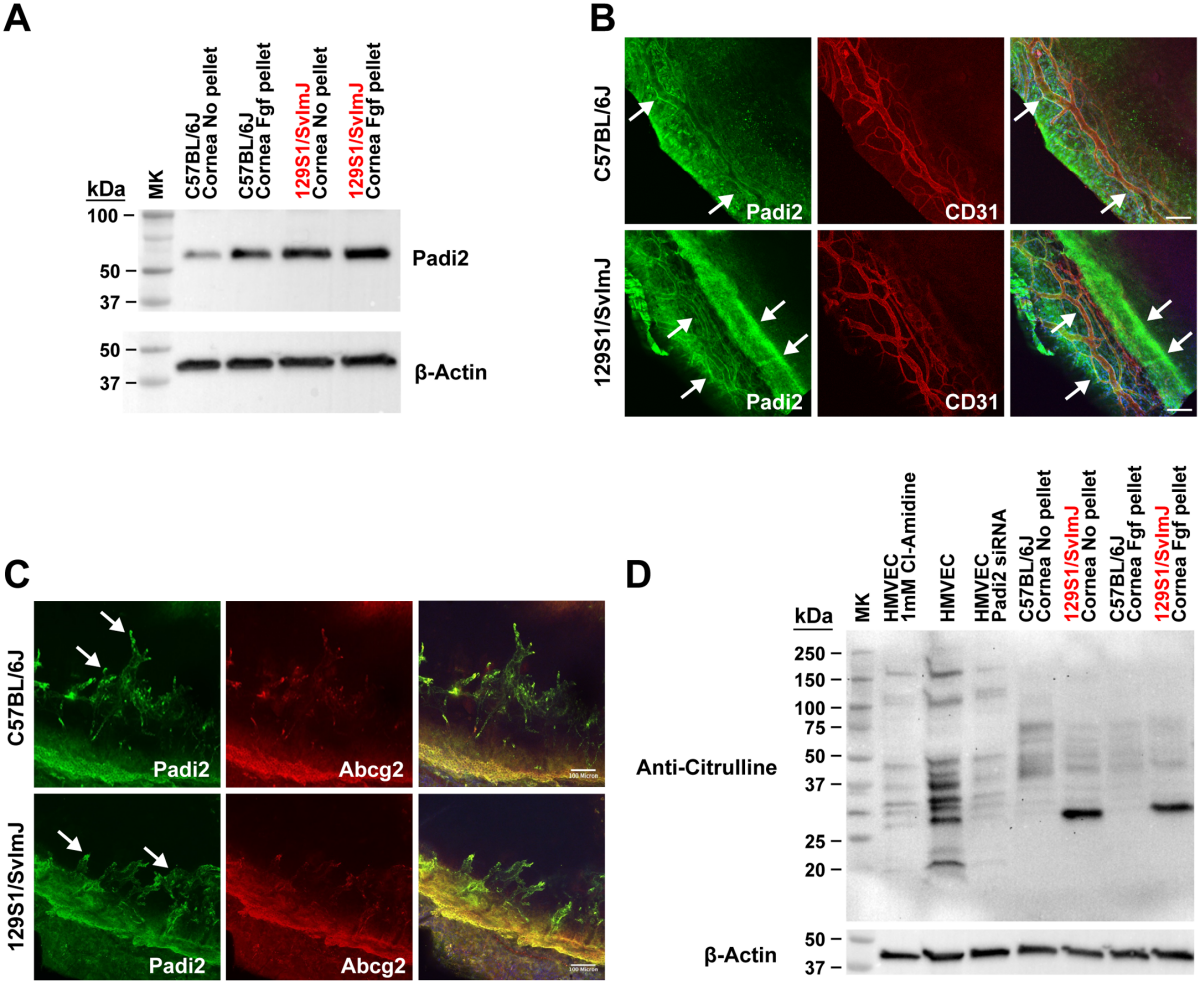
Padi2 expression level corresponds with the level of citrullinated protein in both unstimulated and bFGF stimulated corneas. **A)** Western blot of unstimulated cornea from C57BL/6J and 129S1/SvImJ verifying the RT-PCR experiments. **B)** Immunofluorescent staining of unstimulated corneas from C57BL/6J and 129S1/SvImJ. Quantification of Padi2 staining relative to cell number (S5 Fig) is higher, as visible from the green staining, at the limbal basal epithelial cells and limbal vasculatures of cornea from 129S1/SvImJ strain. **C)** Co-staining experiment for Abcg2 and Padi2 using confocal laser scanning microscopy of bFGF stimulated corneas from C57BL/6J and 129S1/SvImJ. Padi2 is clearly expressed in both Abcg2^+^ limbal basal cells and new bFGF-induced vessels. Scale bar = 100 μm. **D)** Knockdown of PADI2 and inhibition by Cl-Amidine treatment significantly reduces deiminated proteins in HMVECs. Significant difference in intrapeptide citrullination between the two strains. Anti-Citrulline antibody (Abcam, Cambridge, MA) used in this experiment does not react with free citrulline or arginine and reacts only with intrapeptidic citrulline regardless of the amino acid sequence. We further verified this by HPLC (S5 Fig).

We selected Chromosome 4 for further investigation based on the location of the two most significant genome-wide association peaks. Within this EMMA-linked region on chromosome 4, there is a gene cluster containing all five peptidyl arginine deiminase (PADI) family isoforms (Fig 4); however, only *Padi2* and *Padi4* are expressed in corneas undergoing the eye assay. We found no difference in *Padi4* expression between the two strains (C57BL/6J and 129S1/SvImJ) bearing different haplotypes at the *Padi4* locus (S4 Fig). Based on the expression data and proximity to the highest-scoring SNP (rs32857122) in the GWAS, we focused on *Padi2* as a possible candidate for a novel angiogenesis-regulating gene. We verified the difference in Padi2 expression between two strains that do not share the same haplotypes, C57BL/6J and 129S1/SvImJ (a “high” angiogenic strain), by both Western blot and whole mount immunofluorescent labeling (Fig 5A and 5B). Notably, when bFGF stimulated corneas of 129S1/SvImJ mice were compared to C57BL/6J, there was a greater increase in expression of *Padi2* in the 129S1/SvImJ corneas (Figs 3 and 5A). To further determine the cell types expressing Padi2 in the mouse cornea, we used double labeling immunofluorescent staining with limbal epithelial stem cell marker, Abcg2 [23]. Interestingly, we found that Padi2 is mainly expressed in Abcg2^+^ limbal basal cells (Fig 5B) and in endothelial cell of the bFGF-induced vessels (Fig 5B and 5C). We next assessed the level of deiminated proteins (a byproduct of Padi enzymatic activity) in the protein lysates extracted from both the unstimulated and bFGF stimulated corneas (including new vessels but excluding the limbus) of C57BL/6J and 129S1/SvImJ. We found a significant difference in protein deamination level in 129S1/SvImJ cornea samples compared to those of C57BL/6J (Fig 5D). As, *Padi2* is the only differentially-expressed Padi isoform expressed in the cornea, we attribute the large difference in deiminated protein between C57BL/6J and 129S1/SvImJ to elevated Padi2 expression in the “high” angiogenic strain (Fig 5A; we further verified this by HPLC [S6 Fig]). We also evaluated the effect of Cl-Amidine, an inhibitor of PADIs [24], and PADI2 knockdown in human microvascular endothelial cells (HMVECs) and found a significant loss of deiminated proteins in both samples compared to non-treated HMVECs (Fig 5D). These data demonstrate that human endothelial cells exhibit substantial PADI2 enzymatic activity.

### PADI2 inhibition can alter endothelial cell activity *in vitro*

**Fig 6:**
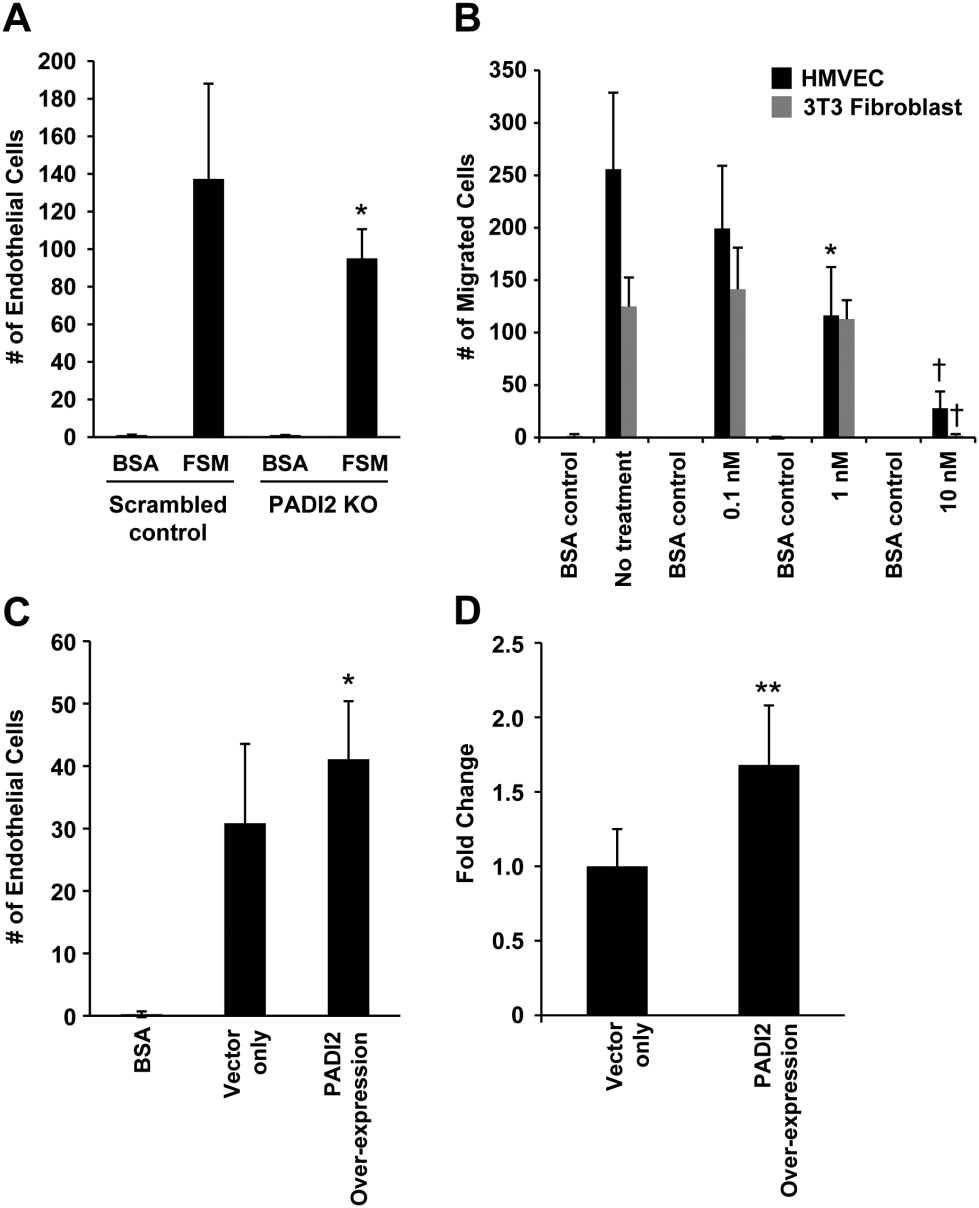
*In vitro* endothelial cell functional assay. **A)** HMVEC migration in response to full serum media (FSM) was significantly decreased when cells were transfected with PADI2-specific siRNA compared to scramble siRNA control. Started with seeded 20,000 cells per well; Basal medium containing 0.1% BSA is used as negative control. We did not find any difference in endothelial cell proliferation (S5 Fig). **B)** HMVEC migration in response to FSM was significantly decreased upon treatment with 1mM Cl-Amidine, a PADI inhibitor, compared to DMSO treated control. We observed no effect in 3T3 fibroblast cell migration when treated with Cl-Amidine. * indicates *P* < 0.05. † Treatment with 10 mM Cl-Amidine was toxic to both cell lines. **C)** HMVEC migration and proliferation (**D**) was assessed when human PADI2 is overexpressed. We observed a significant increase in both endothelial cells migration and proliferation (**D**) when PADI2 is overexpressed compared to vector only transfected cells. Started with seeded 10,000 cells per well. * and ** indicates *P* < 0.05 and *P* < 0.001 respectively.

In order to determine whether PADI2 expression levels affect endothelial cell function, we first assessed the effect of *PADI2* knockdown by siRNA on both proliferation and migration of human microvascular endothelial cells (HMVECs). We observed a significant decrease in the ability of HMVECs to migrate to full serum-containing media in *PADI2* siRNA transfected cells compared to scramble control siRNA transfected cells (Fig 6A). However, we did not observe a difference in HMVEC proliferation between the two groups (S7 Fig). In addition, we treated HMVECs with Cl-Amidine and found a significant decrease in HMVEC migration to full serum-containing media in a dose dependent manner (Fig 6B). Notably, we did not detect an observable effect on 3T3 fibroblast cells when treated with Cl-Amidine, reinforcing the specific role of PADI activity in endothelial cells function. We then transiently overexpressed *PADI2* cDNA (cloned in an expressing vector; pcDNA3.1) in HMVECs and found a significant increase in both migration and proliferation in PADI2 overexpressing cells versus the control vector-only transfected HMVECs (Fig 6C and 6D). These data indicate the potential role of PADI2 and its expression level affects both endothelial cell migration and proliferation.

### Loss of padi2 in zebrafish leads to vascular patterns defects

**Fig 7:**
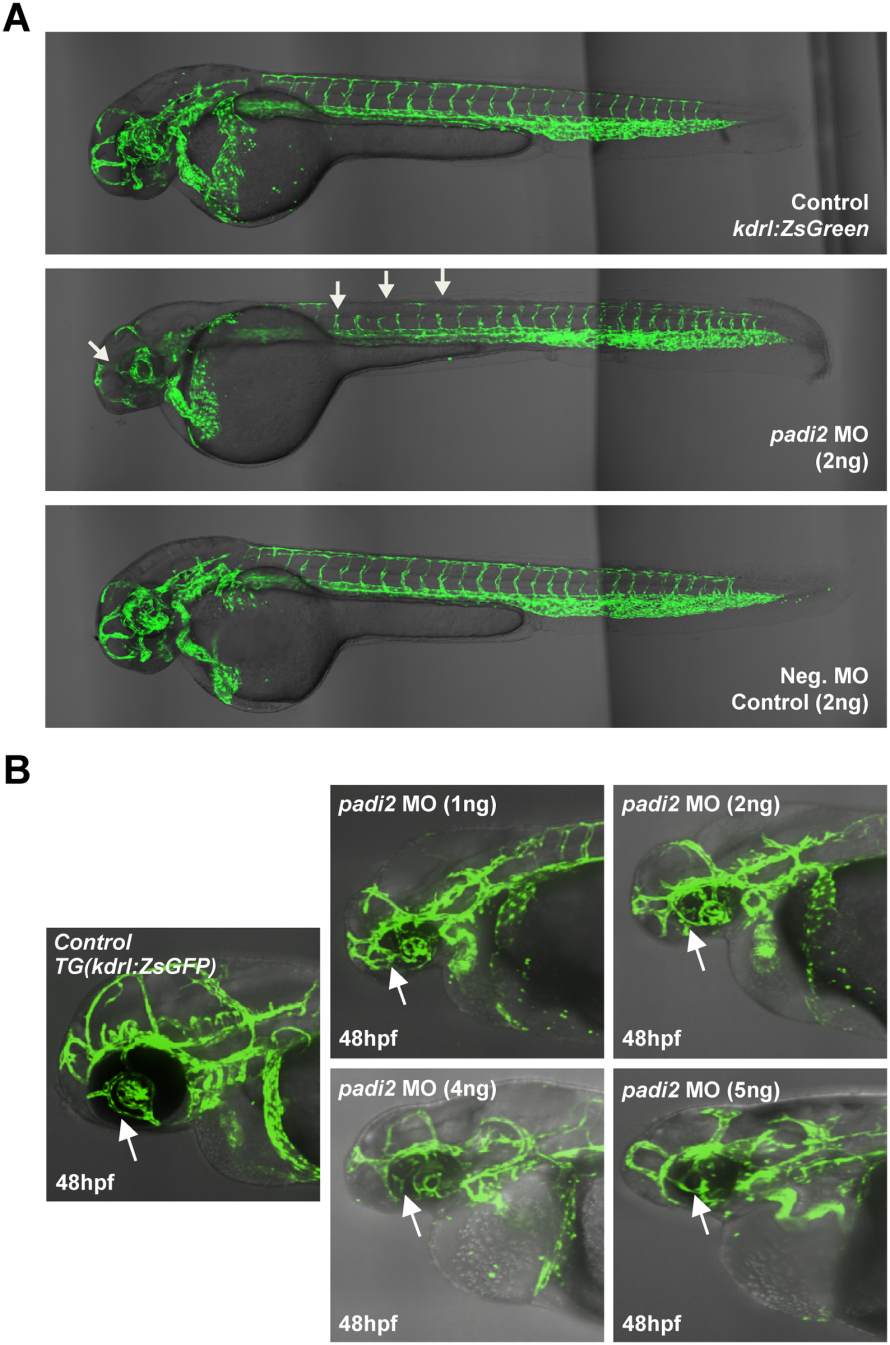
padi2 morphants display vascular defects at 48 hpf. **A)** Significant vascular defects in padi2 MO compared to sham-injected MO (negative) control siblings (representative figures from 16 injections, repeated 4 times). Note the gaps present in the intersegmental vessels suggesting missing or abnormal formation. Also note vessel absence in the head (arrows). Magnification: x10. **B)** Higher magnificantion imgages showing head vascular defects in embryos injected with zebrafish padi2 MO.

**Fig 8:**
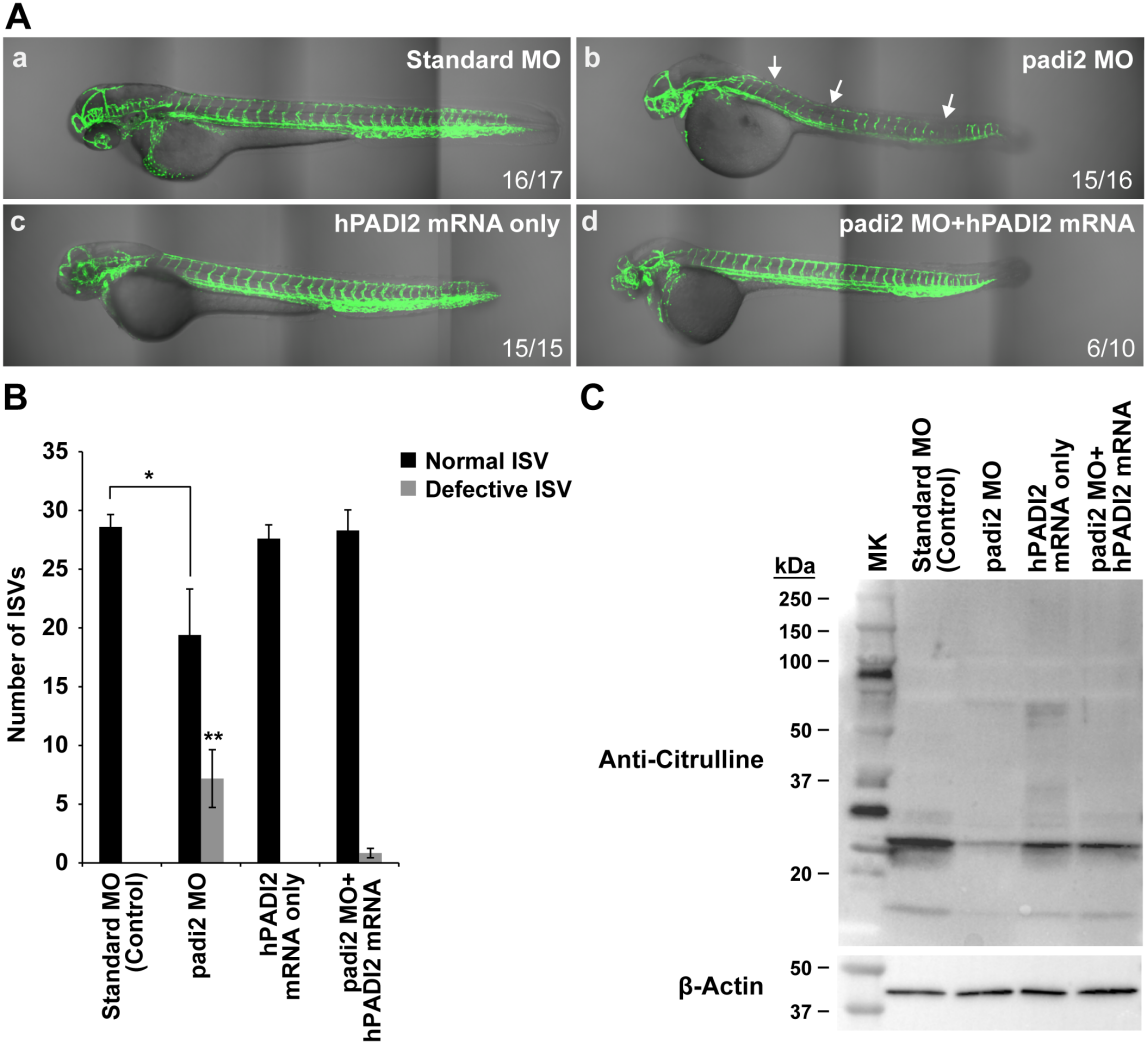
Human PADI2 mRNA can rescue vessel defects in zebrafish padi2 morphants. **A)** Significant vascular defects (arrows) are present in padi2 MO (1.5 ng/embryo) (b) compared to standard (sham-injected) MO control (1.5 ng/embryo) (a) and human PADI2 (hPADI2) mRNA only (225 pg/embryo) (c). Co-injection of hPADI2 mRNA (225 pg/embryo) rescues padi2 MO (1.5 ng) vascular defect. (d) Magnification: x10. **B)** Bar graph of normal and defective ISVs in four different groups, showing average ±SD from four independent experiments. Missing and abnormal formation of ISVs are considered as defective vessels. The number of defective vessels is significantly increased with the injection of padi2 MO (* and ** denotes *P*<0.001 and *P*<0.005 respectively.) **C)** Citrullination levels in zebrafish. The level of deiminated protein is partially rescued in padi2 morphants with the co-injection of hPADI2 mRNA.

To test the effect of padi2 knockdown *in vivo*, we designed a translation-blocking morpholino (ATG-MO, Gene Tools, LLC) targeting the zebrafish *padi2* locus and injected this into transgenic *kdrl*:*zsGreen* zebrafish embryos at the one cell stage (2 ng/embryo injection). We observed defects in vessels in brain, eye and gaps in the formation of intersegmental vessels (ISVs) around 48 hours post fertilization (hpf) (Fig 7A). The observable vascular defect was dose-dependent and a more severe phenotype was observed in higher doses at both 48 and 72 hpf (S8 and S9 Figs). Although morphology defects in head development can be seen, the pattern of head vessels is significantly different between control embryos and morphants (Fig 7B). To further confirm the specific phenotypes observed in padi2 ATG-MO morphants, we targeted *padi2* with a splicing-MO in transgenic *kdrl*:*zsGreen* zebrafish embryos at the one cell stage (3 ng/embryo injection). This MO was designed to target intron6-exon7 splice junction with a complementary sequence to 3’ end of intron 6 (acceptor site) and the 5’-end of exon 7 and aimed to cause aberrant splicing, possibly leaving intron6 in the mature *padi2* mRNA. We confirmed the aberrantly spliced products by RT-PCR (S10A Fig). The phenotypes observed in embryos injected with newly designed MO are consistent with our earlier observations of defects in vessels in brain and intersegmental vessels (ISVs) around 48 hpf (S10B Fig). We also performed rescue experiments by co-injecting padi2 ATG-MO with *in vitro*-generated capped human PADI2 mRNA (GenBank Accession: NM_007365) in order to demonstrate padi2 null morphant phenotypes were due specifically to padi2 down-regulation. At the nucleotide level, the N-terminal human *PADI2* mRNA significantly differs from zebrafish *padi2* and we do not expect the designed padi2 ATG-MO to effectively bind to the human *PADI2* mRNA start site or blocking it from translation since there are many mismatches (14/25; 56%) between the two sequences (S11A Fig). Strikingly, the injection of high copy number of human *PADI2* mRNA alone results in significant toxicity in zebrafish (S11B Fig). By titrating the dose of human *PADI2* mRNA, we determined that injection of up to 225 pg into early embryos did not cause any phenotypic changes or lethality (S11B Fig). For rescue experiments, we then co-injected human PADI2 mRNA (225 pg/injection) with the padi2 MO in early embryos and found that it not only partially rescued the vascular defect observed in padi2 morphants at 48 hpf (from average number of defective ISVs of 7.18± 2.46 to 0.83 ± 0.70 per rescued zebrafish) (Fig 8A and 8B), but also increased the level of deiminated proteins in the rescued zebrafish (Fig 8C), providing evidence for the specificity of padi2 MO and conservation of function between human and zebrafish padi2. Together, these data represent compelling *in vivo* evidence implicating PADI2 function in angiogenesis regulation.

## Discussion

In this study, we took the innovative step of utilizing the variation among common inbred mouse strains to screen for genes affecting angiogenesis. We have previously shown that there is a 10-fold range of response to growth factor-stimulated angiogenesis using the corneal micropocket assay among different inbred strains, and demonstrated that this trait is genetically controlled by different loci [7]. The phenotype distribution and previous linkage based mapping results both indicate that the genetic effect is not the result of a single polymorphic gene. Rather, angiogenic responsiveness is controlled by the sum of the effects of multiple polymorphisms with both positive and negative effects [8, 9].

The recent dense genotyping of 94 inbred mouse strains, including 73 classical inbred strains, has enabled the imputation of genotypes across the bulk of their genome in all these strains [17, 25]. As a result, we are able to make greater use of both SNP-based association mapping and haplotype structure to identify specific base-pair changes that affect angiogenic responsiveness across a wider variety of strains, and a correspondingly wider variety of genomic diversity. By sampling naturally occurring genetic variation, it allows us to discover novel angiogenesis-regulating genes and pathways. The large number of recombinations due to the breeding history of classical mouse strains has resulted in a dramatic reduction in the number of candidate genes in a given region, significantly accelerating fine-mapping. In order to have a high statistical power and avoid false-positives for genome-wide association mapping, we phenotyped a set of 42 inbred strains [21, 26–28]. With a broad and diverse range of measured vessel area among the tested strains, we were able to map 5 novel angiogenesis-response QTLs corresponding to 47 candidate genes. By examining the available sequence of each gene for SNPs that may alter amino acid sequences, or gene expression correlation with regions containing the same type of variant among inbred mice who have “high” or “low” angiogenic phenotype, we further narrowed our list of candidates to 14 genes. Of these, two genes, *Irf2bp2* (Interferon regulatory factor 2 binding protein 2, PMID: 615332) and *Slc38A1* (Solute Carrier Family 38, Member 1, PMID: 608490), were already implicated in the regulation of angiogenesis [29, 30], serving as validation of our approach. The other candidate genes are novel to angiogenesis and previously known to be involved in metabolic, signaling and structural pathways. While all these candidate genes warrant further evaluation in their role in angiogenesis, we selected Padi2 for this study based on the location of the two highest significant genome-wide association peaks on chromosome 4 where it spans the Padi isoform family (rs32857122; p = 9.34 x 10^−7^ and rs32259427; 3.67 x 10^−6^; Table 1).

Previous studies have shown that PADIs are involved in many physiological processes such as metabolic pathways, cell differentiation, embryonic development, and gene regulation [18]. PADIs are a family of posttranslational modification enzymes that deiminate positively-charged arginine residues on substrate proteins to neutrally-charged citrulline in the presence of Ca^2+^ [18]. This results in an increase of protein hydrophobicity that could potentially affect the structure and ultimately the function of substrate proteins [18]. The expression of PADI isoforms is generally tissue specific; PADI1 and PADI3 are found in the skin epidermis and hair follicles, respectively, while PADI6 is expressed in eggs and embryos [18, 31]. PADI2 is widely expressed, while PADI4 is the only isoform that is translocated to the nucleus and mainly expressed in hematopoietic cells [19].

In the present study, we found that among common inbred mouse strains *Padi2* expression strongly correlates with both cis-genomic variation and with the extent of angiogenic response in “low” or “high” strains. We also found by immunofluorescent staining that Padi2 is expressed in both the limbus and bFGF-induced vessels. By double immunofluorescent staining, we observed that Padi2 is strongly expressed in Abcg2+ limbal basal cells that are enriched with stem cell properties. We next evaluated its role in angiogenesis by *in vitro* functional assays in human microvascular endothelial cells. Knockdown of PADI2 by siRNA and or inhibition by Cl-Amidine treatment in HMVECs led to significant decreases in migration. In contrast, over-expression of PADI2 increased both migration and proliferation in endothelial cells, strongly suggesting the ability of a PADI2 expression and functional activity to alter angiogenic function. Our *in vivo* functional modeling in zebrafish supported the role of padi2 in angiogenesis; suppression of *padi2* expression by blocking translation or normal splicing in zebrafish caused vascular defects in both eye vessels and intersegmental vessels during early development. Complementation of *padi2* suppression with human *PADI2* significantly rescued the vascular defects in *padi2* morphants, indicating the specificity of the morphant phenotype and conservation of function between human and zebrafish *padi2*. Remarkably, injection of high copy numbers of human *PADI2* mRNA alone resulted in toxicity in zebrafish.

An immediate question remains regarding the mechanism by which Padi2 impacts angiogenic responsiveness. PADIs have distinct substrate specificities regardless of their subcellular distribution [32, 33]. Known substrates for Padi2 include MBP (myelin basic protein) in the central nervous system, vimentin in skeletal muscle and macrophages, and β/γ-actin in HL-60 cells [32, 34, 35]. Padi2 appears to have different substrates in various tissues and there is a balance between both its expression and the level of its targeted proteins. Its high overexpression can have pathological consequences that arise from detrimental levels of citrullinated proteins. Previous studies have shown *Padi2* overexpression in transgenic mice under the regulation of the mouse MBP promoter (expressing up to 15 copies of transgene) is toxic to the animal and results in myelin loss in the central nervous system [36]. It has also been shown that *Padi2* overexpression in skin promotes spontaneous skin neoplasia in mice [37]. A recent paper examining the role of Padi2 in mast cells generated a Padi2 mouse knockout model which did not have a grossly obvious vascular phenotype [38]. However, Padi2 knockout mice would not be expected to show significant vascular defects in the C57BL/6J background. As we show, the difference between the high Padi2 expression in the 129S1/SvImJ mice and low expression in the C57BL/6J mice accounts for the contribution of this locus to the angiogenic phenotype (Figs 3 and 5). Thus knocking out Padi2 in the C57BL/6J background would not be informative for vascular studies since the levels are already very low. An additional complication of studying Padi2 mouse knockouts is that since mice express five different Padi isozymes, there is the potential for partial compensation from the other Padi isozymes [39].

In summary, our GWAS screen in mice has identified 5 novel loci that could affect angiogenic responsiveness. These genes and others in associated pathways can not only serve as candidate genes for clinical association studies, but also provide new therapeutic targets in a wide variety of systemic angiogenesis-dependent diseases. Our study is the first to demonstrate the potential role of *Padi2* as an angiogenesis-regulating gene. We show comprehensive *in vitro* causal evidence of *Padi2* involvement in endothelial cell migration and proliferation. In addition, we report *in vivo* supporting causal evidence of *padi2* involvement in zebrafish vasculature system. Further studies to elucidate the role of Padi2 in both stem cells and endothelial cells may provide valuable new information in understanding the genetic heterogeneity of angiogenic responsiveness and how this heterogeneity correlates with the susceptibility to angiogenesis-dependent diseases such as cancer, multiple sclerosis, rheumatoid arthritis, and macular degeneration.

## Materials and methods

### Mouse strains and corneal micropocket assay

All mouse strains (male, ~ 7–9 weeks old): 129S1/SvImJ, 129T2/SvEms, 129X1/SVJ, A/J, AKR/J, Balb/cByJ, Balb/cJ, BPH/2J, BPL/1J, BPN/3J, BUB/BnJ, CE/J, C3H/HeJ, C57BL/6J, C57BL/10J, C57BLKS/J, C57BR/cdJ, C58/J, CBA/J, DBA/1J, DBA/2J, DDY/JclSidSeyFrk, FVB/NJ, I/LnJ, KK/HlJ, LG/J, LP/J, MA/MyJ, MRL/MpJ, NOD/LtJ, NON/LtJ, NOR/LtJ, NZB/BlNJ, NZL/LtJ, NZW/LacJ, P/J, RIIIS/J, SEA/GnJ, SJL/J, SM/J, SWR/J, TALLYHO/JngJ were obtained from Jackson Laboratories (Bar Harbor, ME, USA) and housed in Boston Children’s Hospital’s animal facility. The corneal micropocket assay was performed as previously described [20]. Briefly, a slow-release pellet containing 20ng of bFGF (R&D Systems, Minneapolis, MN, USA) was implanted in the mouse cornea ~1.0 mm from the limbus. The area of vascular response was assessed on the fifth postoperative day using a slit lamp. Vessel area was calculated using the equation 0.2π x VL x CH, where VL is vessel length from the limbus in millimeters and CH is clock hours around the cornea. Five mice (10 eyes) per strain were analyzed and similar numbers of control C57BL/6J were included in each assay to confirm consistency. In all experiments, each eye was treated as an independent measurement and the data are reported as the mean ± standard deviation. All animal studies were conducted according to protocols approved by the Institutional Animal Care and Use Committee of Boston Children’s Hospital (approval number 15-08-2998R and 14-10-2789R for mouse and zebrafish experiments respectively).

### EMMA-GWAS

To correct for population structure and genetic relatedness among inbred strains, we used the efficient mixed model association (EMMA) method to assess association of potential SNPs to angiogenic response [21]. The EMMA algorithm was applied in the R statistical package using vascular area data obtained from 42 inbred strains. As detailed previously [21], adjusted association p-values were calculated for 132k SNPs with minor allele frequency of > 5% (p<0.05 genome-wide equivalent for genome-wide association in common inbred strains is set at p = 4.1 X 10^−5^ or -log10P = 4.39). Known and imputed dense SNP data were downloaded from http://phenome.jax.org/db/q?rtn=snps/download.

### Total RNA/Protein extraction and expression analysis

Corneas harvested from each mouse were immediately stored in *RNAlater* RNA Stabilization reagent (Qiagen, Valencia, CA, USA). Each cornea was trimmed excluding the limbus and homogenized using PRO200 homogenizer (PRO Scientific, Oxford, CT, USA). Total RNA was isolated from dissected corneas using RNeasy Kit (Qiagen, Valencia, CA, USA). Total RNA of 5ug for each sample was reverse transcribed with the Superscript First-strand cDNA synthesis kit (Invitrogen, Carlsbad, CA, USA) using a random primer. Quantitative real-time PCR (qRT-PCR) for each gene (listed in S2 Note) was performed in triplicate using TaqMan Gene Expression assays (Life Technologies, Grand Island, NY, USA). The relative level of each RNA sample is calculated using the ΔΔCt method normalized to the corresponding housekeeping gene *Gapdh* and 18S rRNA levels. Student’s *t*-test comparing C57BL/6J as reference control and different strains were performed. Statistical significance was defined as *P*<0.05. For protein extraction, cornea samples were trimmed above the limbus and then added to the appropriate amount of T-PER Tissue Protein Extraction Reagent (Thermo Fisher Scientific, Rockford, lL, USA) containing Thermo Fischer Scientific Halt Protease Inhibitor Cocktail, EDTA-Free (Product No. 87785). After homogenization, total sample proteins were determined using Bicinchoninic Acid Assay (BCA). Total of 15–20μg of protein samples were mixed with loading dye (6X, SDS sample buffer, Boston BioProducts, Boston, MA, USA), heated to 100°C for 5 minutes, and then loaded on a 4–20% gradient SDS PAGE gel (Bio-Rad, Hercules, CA, USA) It was then resolved and electro-blotted onto a PVDF membrane. Blots were blocked with 3% non-fat milk and incubated overnight at 4°C with the appropriate antibody (primary antibodies and dilutions are listed in S3 Note). The blots were then washed, incubated with a 1:10,000 dilution of corresponding HRP-conjugated secondary antibody, and labeled proteins were detected using enhanced chemiluminescence.

### Whole mount double immunostaining

Eyes from C57BL/6J and 129S1/SvImJ adult male mice were fixed in 4% PFA for 1 hour and transferred to phosphate buffered saline. Corneas were dissected to include the limbus and whole mount immunostaining was carried out using antibodies against Padi2 (Abcam, Cambridge, MA, USA), Abcg2 (Abcam, Cambridge, MA, USA) and CD31 (BD BioScience, San Jose, CA, USA) as previously described [22]. Anti-rabbit IgG and anti-mouse IgG secondary antibodies were labeled with Alexa Fluor 488 Dye (Invitrogen, Carlsbad, CA, USA) or Alexa Fluor 555 dye (Invitrogen, Carlsbad, CA, USA), respectively. Corneas were flattened by making 4 radial cuts and mounted using ProLong Diamond Antifade mountant with DAPI (Invitrogen, Carlsbad, CA, USA) on a glass slide (epithelium up) and sealed under a coverslip. They were imaged on Leica SP2 inverted confocal microscope using 20x and oil immersion 63x objectives. Padi2 staining was quantified using ImageJ and measured expressed relatively to cell number (DAPI staining). Three images were taken for each cornea sample.

### Gene silencing and *in vitro* functional assays

Human microvascular endothelial cells-dermal (HMVEC-d) (Lonza, Walkersville, MD, USA) were cultured in EGM-2MV BulletKit (Lonza, Walkersville, MD, USA) according to the vendors’ instructions and used at early passages (before passage 5). HMVECs were seeded in 60mm plates and transfected with high grade quality flexitube Genesolution siRNA for PADI2 (PMID: NM_007365; Qiagen, Valencia, CA, USA) using Lipofectamine RNAiMAX (Life Technologies, Grand Island, NY, USA) according to the manufacturer’s protocol. As negative control we used non-targeting control siRNA, which has no known target mRNA and is shown to have no effect on the cells (Negative Control siRNA, Qiagen, Valencia, CA, USA). Protein knockdown was assessed by western blot 72 hours after transfection. The 3T3 fibroblast cell line was obtained from ATCC (Manassas, VA, USA) and cultured according to manufacturer’s directions. Where indicated, Cl-Amidine (EMD Millipore, Billerica, MA) was diluted in cell culture medium at the final concentration of 0.1 mM, 1 mM and 10 mM and added to cells for 24 hrs prior to functional assays. For migration assay, polycarbonate transwell inserts, 6.5mm diameter with 8.0μm pores, were coated with 50μL fibronectin (20μg/ml) (BD BioScience, San Jose, CA, USA). Cells were harvested and resuspended in EBM-2 (Lonza, Walkersville, MD, USA) containing 0.1% BSA (Bovine Serum Albumin) (Thermo Fisher Scientific, Rockford, lL, USA). Each well was plated with 20,000 cells containing full serum media or EBM-2 containing 0.1% BSA as negative control. Cells were allowed to migrate for 4 hours. Cells on the top of the membrane were removed using cotton-tipped applicators and membranes were processed using Diff-Quick (Dade diagnostics, Aguada, PR). Following staining, membranes were rinsed in PBS and removed from the insert using a scalpel. Membranes were then mounted on slides, and the number of cells in a microscopic field was counted manually. For proliferation assay, HMVEC-d cells were plated at 2,000 cells/ml into 96-well culture plates containing EGM-2MV BulletKit (Lonza, Walkersville, MD, USA). Cells were incubated at different time points (24, 48 and 72 hours). Cell proliferation was evaluated using the CyQUANT Cell Proliferation Assay Kit (Molecular Probes. Eugene, OR, USA). Fluorescence measurements were made using a microplate reader with excitation at 485 ± 10 nm and emission detection at 530 ± 12.5 nm. Student’s *t* tests comparing wild type controls and siRNA treated cells were performed. Statistical significance was defined as *P*<0.05.

### High-Performance Liquid Chromatography (HPLC)

Twenty micrograms of protein samples were analyzed by reverse phase -HPLC (RP-HPLC). Briefly, the samples were dissolved in Solvent A containing H_2_O/CH_3_CN/TFA (99/1/0.10), and 100μl of the sample was injected on to a 5 μm, 9.4 mm x 250 mm RP-HPLC column (Agilent Technologies, Santa Clara, CA) and eluted at 2.5 ml/min with a gradient of 5%-95% solvent B containing CH_3_CN/H_2_O/TFA (90/10/0.07) for 20 minutes. The samples were visualized at 280 nm and eluents were collected in three factions over a 20 minutes period. The samples were lyophilized and tested for presence of proteins of interest using polyclonal citrulline antibody (Abcam, Cambridge, MA) in western blot analysis as previously described.

### Zebrafish maintenance, morpholinos and microinjection

Zebrafish were maintained and handled according to our vertebrate animal protocol that has been approved by Boston Children’s Hospital Animal Care Committee (approval number 14-10-2789R) and includes detailed experimental procedure for all *in vivo* experiments described in this paper. Zebrafish embryos were cultured in “egg water” consisting of 0.03% sea salt and 0.002% methylene blue. Morpholinos (MOs) were injected into embryos at one-cell stage. Morpholino oligonucleotides were designed by and ordered from Gene Tools (Philomath, OR, USA). The morpholino sequences used for “ATG start site” and “intron 6-exon 7 splice junction” target of padi2 are 5’- TAAGAGATCGACGGGACACCATGAT-3’ and 5’- GTACTCTGATGCACGAAATAGGAAC-3’ respectively and the negative control (standard morpholino) is 5’- CCTCTTACCTCAGTTACAATTTATA-3’. For mRNA synthesis, human PADI2 cDNA was purchased from Harvard PlasmID Database (Boston, MA, USA). The cDNA sequence was verified by the Harvard PlasmID core facility. Capped human PADI2 mRNA was transcribed from linearized plasmid using mMESSAGE mMACHINE T7 Transcription Kit (Ambion, Austin, TX, USA), purified and diluted for injection of embryos at one-cell stage. The RT-PCR primers used in this experiment is listed in S4 Note.

## Supporting information

S1 Fig QQ-plot for the GWAS on vessel area from 42 common inbred strains. QQ-plots of observed vs. expected LOD scores. Left is an uncorrected plot showing artifactually inflated LOD scores resulting from population structure. On the right is the EMMA-corrected plot.
(TIF)

S2 Fig Gene expression of *Slc38A1* (a candidate gene) within the statistically significant region identified by EMMA in mouse Chr. 15. No significant expression differences of *Slc38A1* among inbred strains with different haplotypes. Each color indicates a haplotype.
(TIF)

S3 Fig Nonsynonymous SNP (rs50093952) in mouse Chr. 15. Nonsynonymous SNP (only observed in “high” angiogenic strains) where the derived allele changes an amino-acid residue is conserved in all mammalian species. The image is taken from UCSC Genome Browser on Mouse July 2007 (NCBI37/mm9) Assembly.
(TIF)

S4 Fig Padi4 expression level. Western blot of unstimulated cornea from C57BL/6J and 129S1/SvImJ. We found no differences in Padi4 expression between the two strains.
(TIF)

S5 Fig Quantification of Padi2 staining. Padi2 expression is quantified relative to cell number and compared between the two strains of 129S1/SvImJ and C57BL/6J. Three images were taken from four independent cornea samples. * indicates *P* < 0.05; two-sided Student’s *t*-test.
(TIF)

S6 Fig RP-HPLC- Unstimulated cornea samples were immunoprecipitated using Citrulline antibody (Abcam, Cambridge, MA), then digested and analysed by MALDI/MS-MS. We clearly observe a difference in citrullinated protein in 129S1/SvImJ cornea compared to C57BL/6J (circled).
(TIFF)

S7 Fig No change in proliferation of HMVECs when PADI2 is knockdown. HMVEC proliferation did not change significantly when cells were transfected with PADI2-specific siRNA compared to scramble siRNA control. Started with seeded 10,000 cells per well. *P* < 0.05; two-sided Student’s *t*-test.
(TIF)

S8 Fig *padi2* morphants display vascular defects at 48 hpf in a dose dependent manner. Gaps in the formation of intersegmental vessels (missing or abnormal pattern). Also note structural body malformation at higher doses. Magnification: x10.
(TIF)

S9 Fig *padi2* morphants display vascular defects at 72 hpf in a dose dependent manner. Gaps in the formation of intersegmental vessels (missing or abnormal pattern). Also note structural body malformation at higher doses. Magnification: x10.
(TIF)

S10 Fig Splicing padi2 morphants display vascular defects at 48 hpf. **A)** RT-PCR of total RNA extracted from padi2 morphants show the newly designed MO causes an aberrant splicing resulting to partial retention of intron 6 within the transcript. **B)** Significant vascular defects in a new padi2 MO compared to standard-injected MO (negative) control siblings (representative figures from 12 injections, repeated 3 times). Note the gaps present in the intersegmental vessels suggesting missing or abnormal formation.
(TIF)

S11 Fig Dosage Titrations for *padi2* MO and *hPADI2* mRNA injection. **A)** Poorly conserved region at the 5’ ATG start site between zebrafish *padi2* and human *PADI2* mRNA sequence. The designed MO targets the ATG start site (highlighted) of zebrafish padi2. MOs differ by more than five out of 25 nucleotides from their target sequence do not interfere with translation of the targeted mRNA. **B)** By titrating the dose of human *PADI2* (*hPADI2*) mRNA, we determined that injection of up to 225 pg into early embryos did not cause any phenotypic changes or lethality. Notably, injection of high copy number of h*PADI2* mRNA (>250 pg) alone results in significant toxicity in zebrafish.
(TIF)

S1 Note Measured vessel area across strains. Vessel area ranges from a value of 0.42 mm^2^ in NZB/BINJ (low angiogenic strain) to 2.05 mm^2^ in AKR/J (high angiogenic strain).
(DOCX)

S2 Note List of Taqman Gene Expression assays. (DOCX)

S3 Note List of primary antibodies and dilutions used for western blot and immunostaining. (DOCX)

S4 Note List of primers used in RT-PCR for zebrafish experiments. (DOCX)
