# Supplementary figures and images for: Identification of Padi2 as a novel angiogenesis-regulating gene by genome association studies in mice
Source: PLoS Genet. 2017 Jun 15;13(6):e1006848. doi: 10.1371/journal.pgen.1006848 (PMC5491319; doi:10.1371/journal.pgen.1006848)

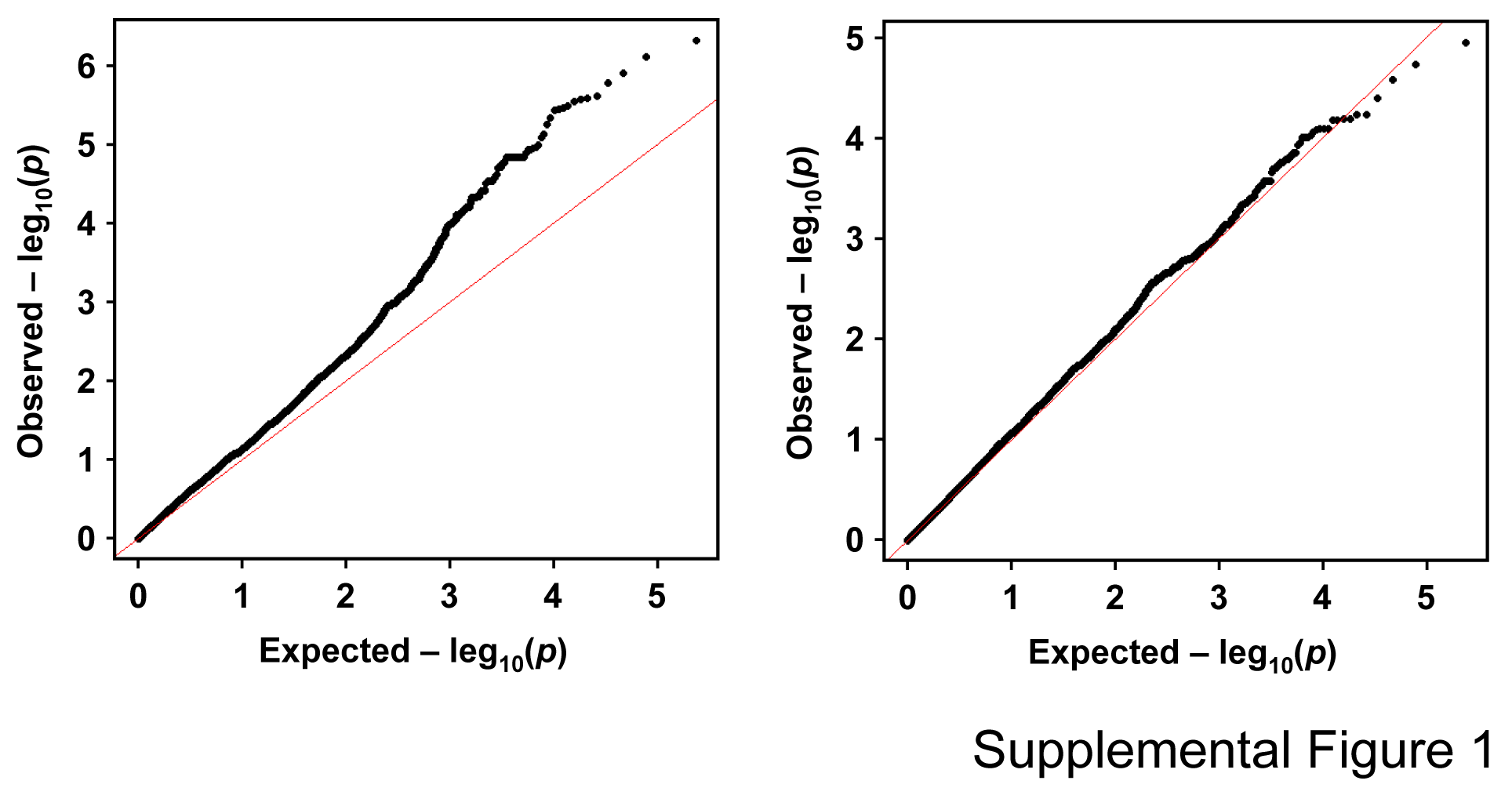

Supplement: S1 Fig — QQ-plots of observed vs. expected LOD scores. Left is an uncorrected plot showing artifactually inflated LOD scores resulting from population structure. On the right is the EMMA-corrected plot. (TIF) [file pgen.1006848.s001.tif]

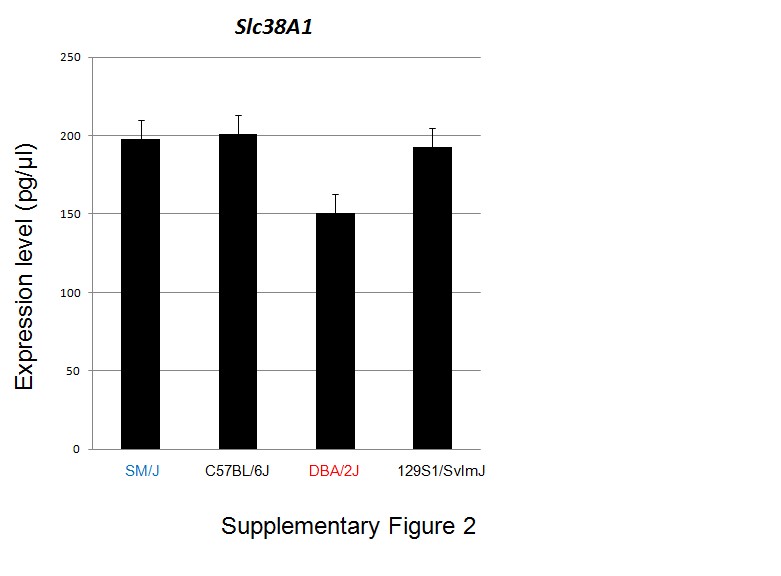

Supplement: S2 Fig — No significant expression differences of Slc38A1 among inbred strains with different haplotypes. Each color indicates a haplotype. (TIF) [file pgen.1006848.s002.tif]

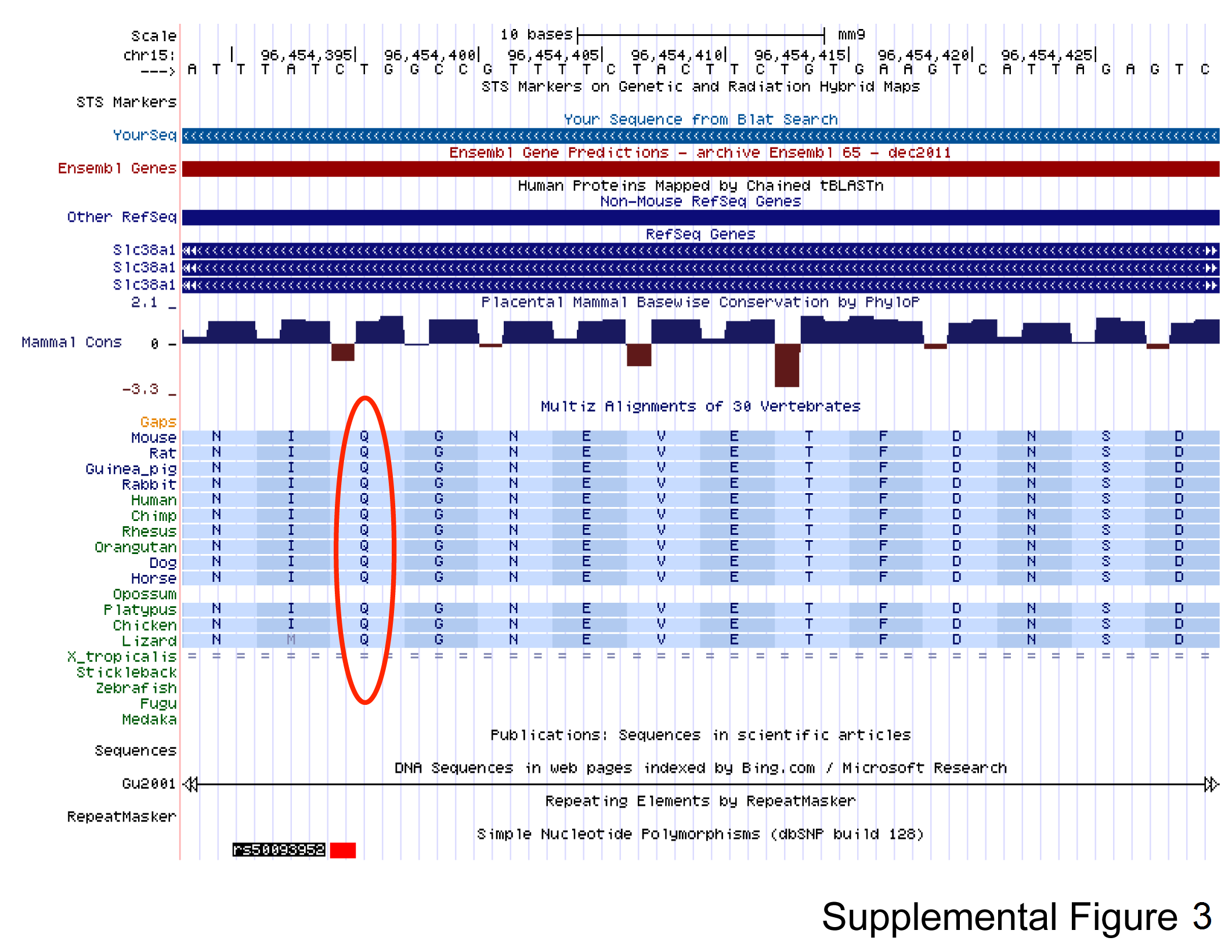

Supplement: S3 Fig — Nonsynonymous SNP (only observed in “high” angiogenic strains) where the derived allele changes an amino-acid residue is conserved in all mammalian species. The image is taken from UCSC Genome Browser on Mouse July 2007 (NCBI37/mm9) Assembly. (TIF) [file pgen.1006848.s003.tif]

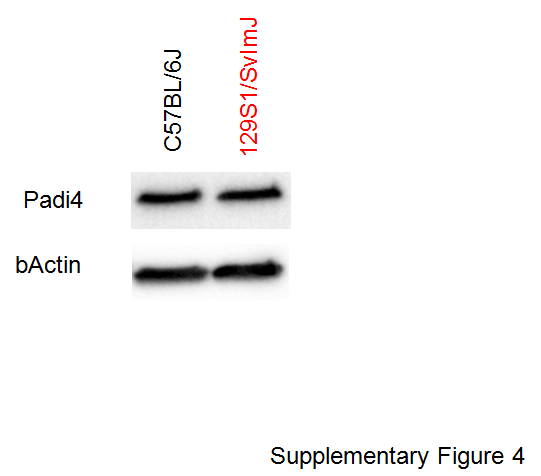

Supplement: S4 Fig — Western blot of unstimulated cornea from C57BL/6J and 129S1/SvImJ. We found no differences in Padi4 expression between the two strains. (TIF) [file pgen.1006848.s004.tif]

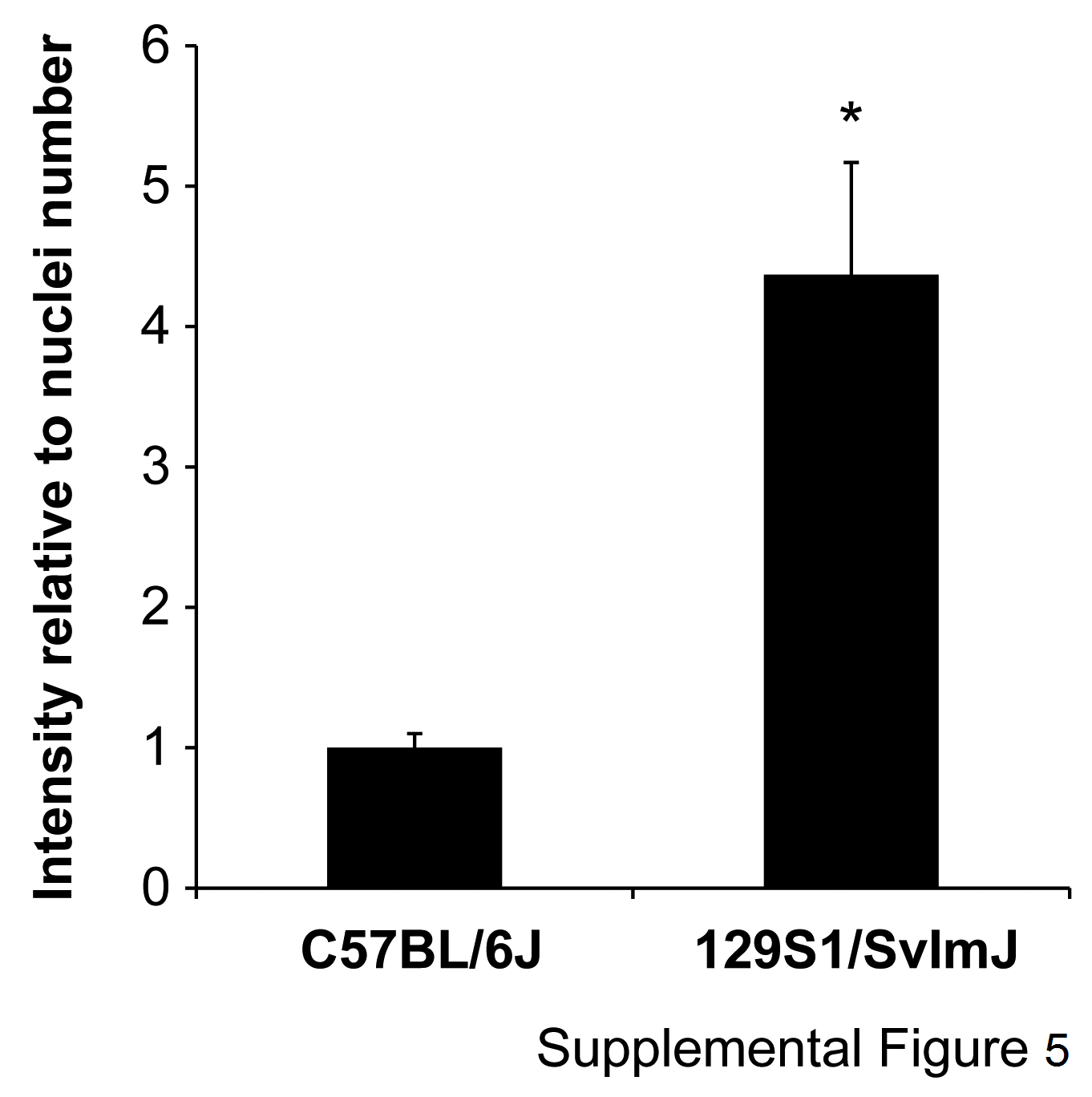

Supplement: S5 Fig — Padi2 expression is quantified relative to cell number and compared between the two strains of 129S1/SvImJ and C57BL/6J. Three images were taken from four independent cornea samples. * indicates P < 0.05; two-sided Student’s t-test. (TIF) [file pgen.1006848.s005.tif]

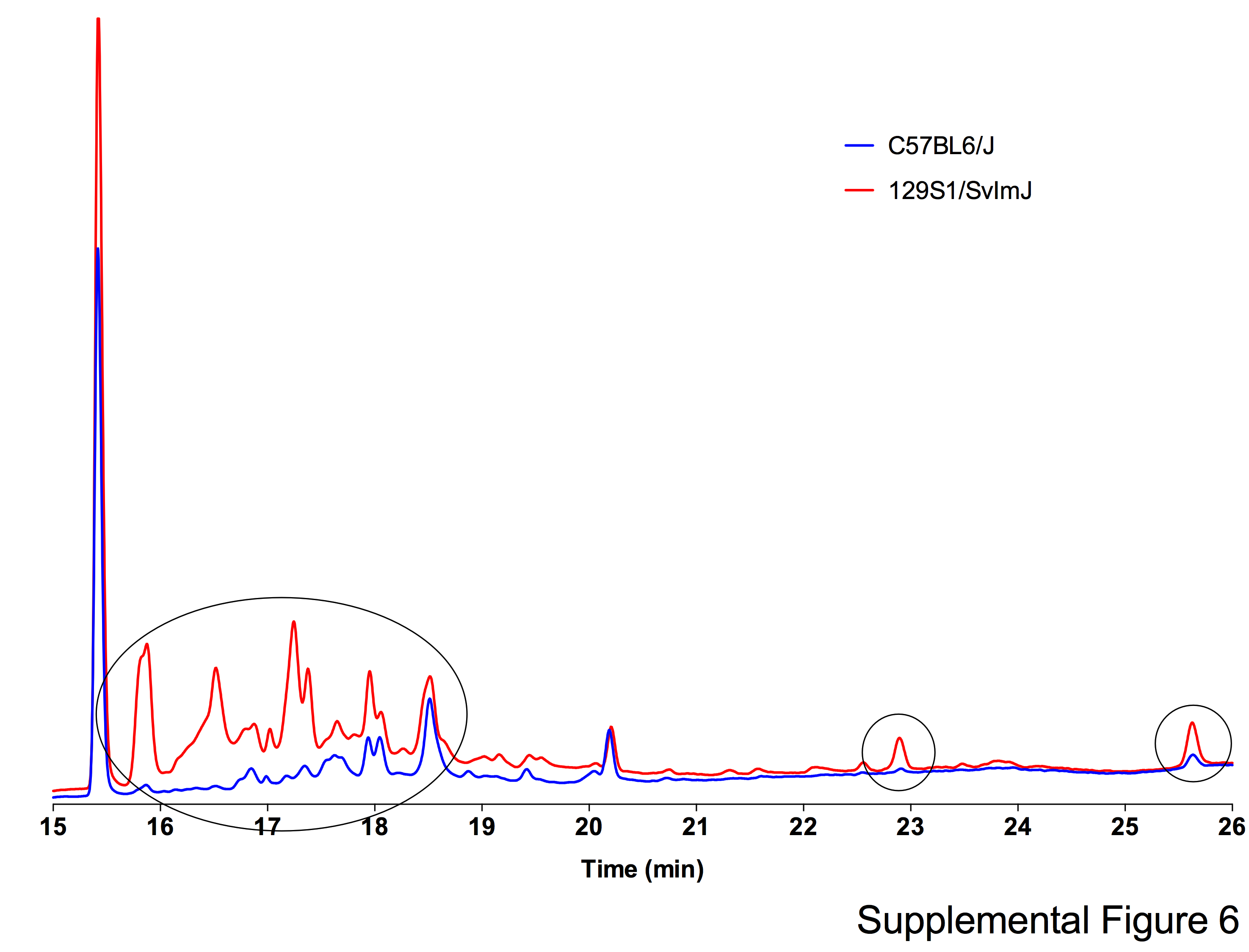

Supplement: S6 Fig — We clearly observe a difference in citrullinated protein in 129S1/SvImJ cornea compared to C57BL/6J (circled). (TIFF) [file pgen.1006848.s006.tiff]

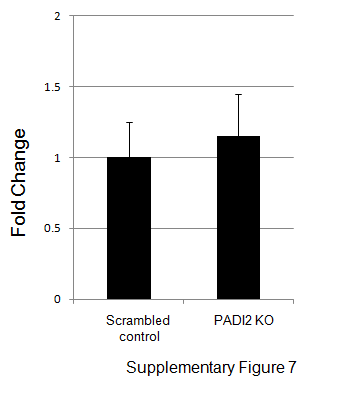

Supplement: S7 Fig — HMVEC proliferation did not change significantly when cells were transfected with PADI2-specific siRNA compared to scramble siRNA control. Started with seeded 10,000 cells per well. P < 0.05; two-sided Student’s t-test. (TIF) [file pgen.1006848.s007.tif]

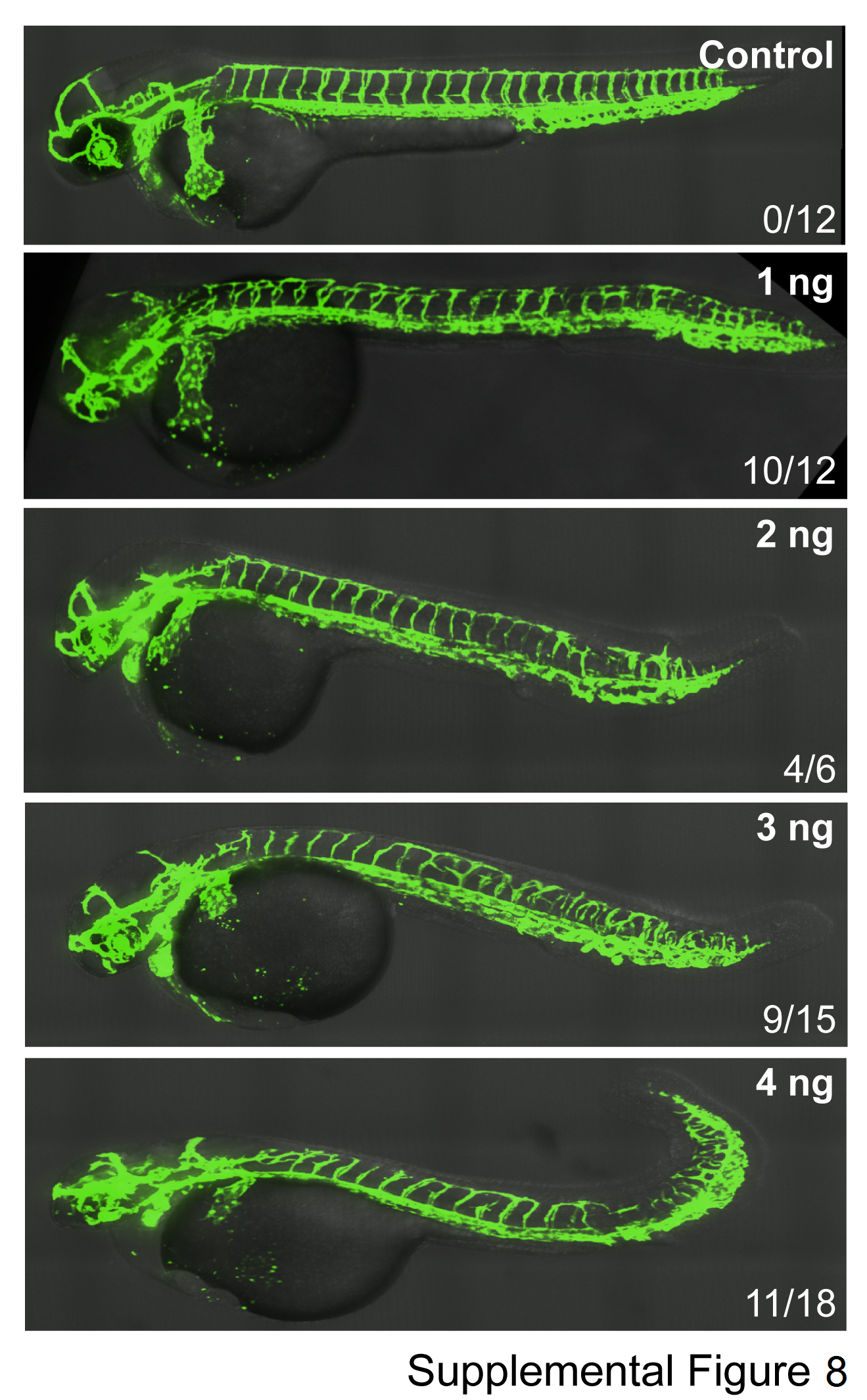

Supplement: S8 Fig — Gaps in the formation of intersegmental vessels (missing or abnormal pattern). Also note structural body malformation at higher doses. Magnification: x10. (TIF) [file pgen.1006848.s008.tif]

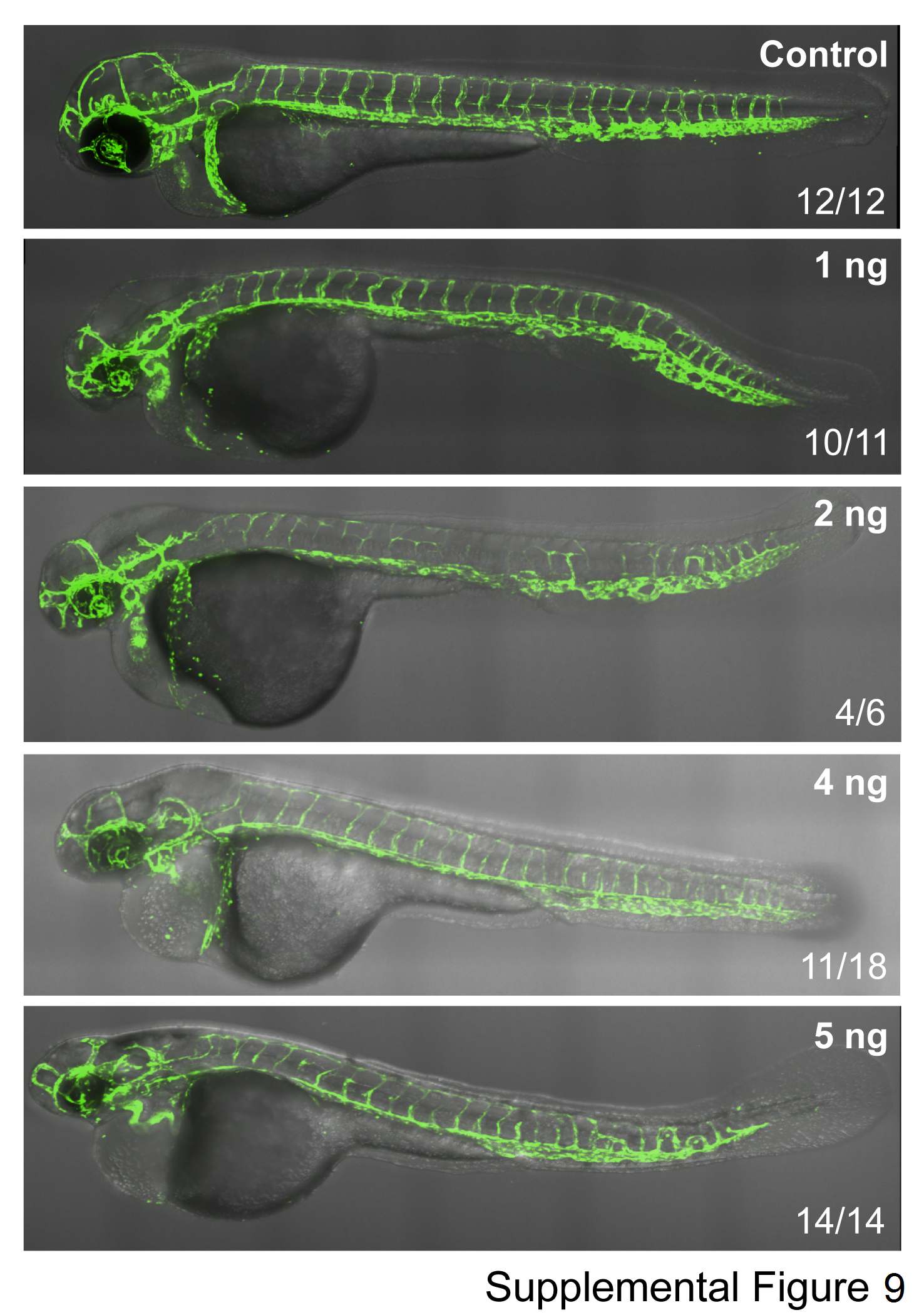

Supplement: S9 Fig — Gaps in the formation of intersegmental vessels (missing or abnormal pattern). Also note structural body malformation at higher doses. Magnification: x10. (TIF) [file pgen.1006848.s009.tif]

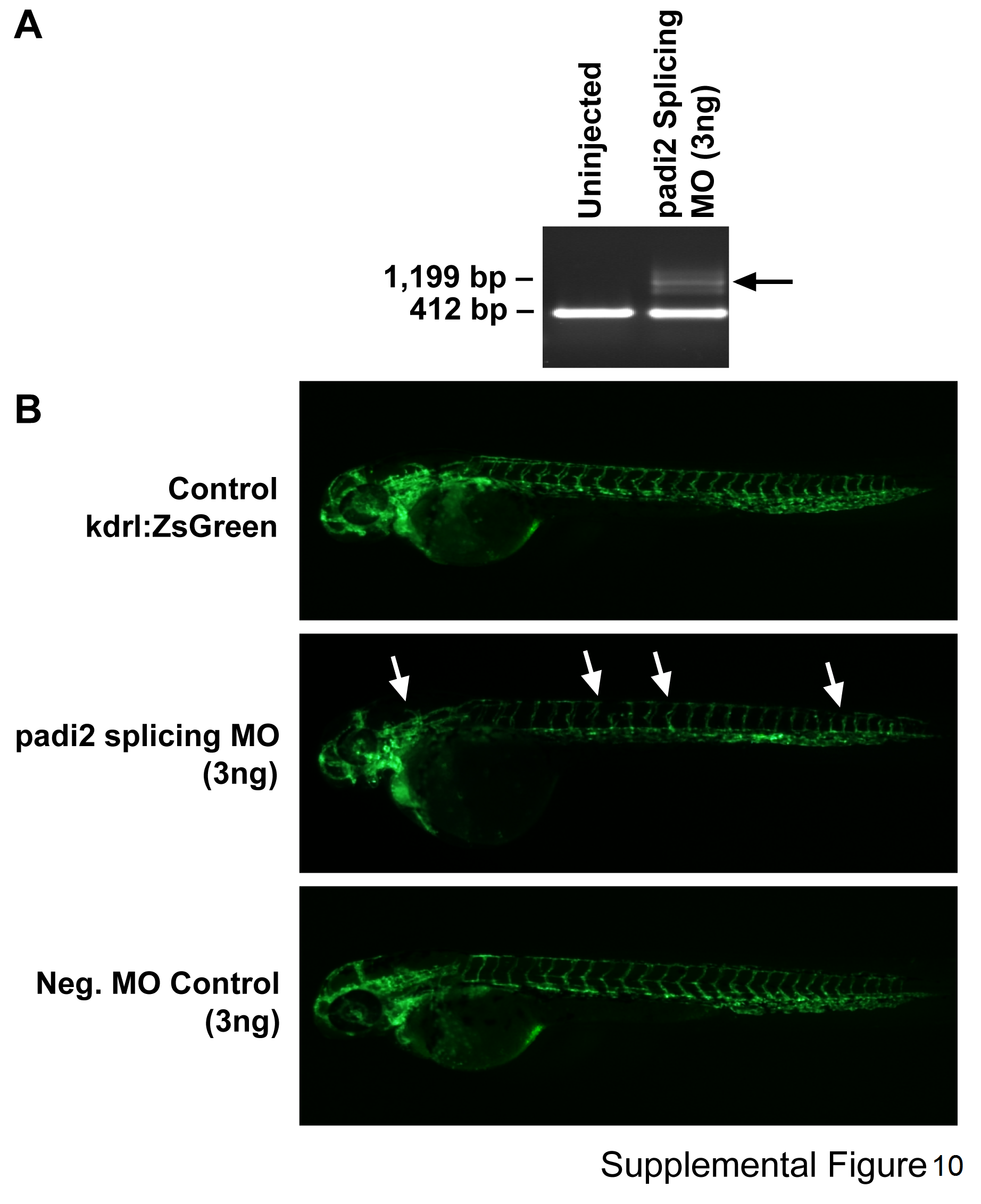

Supplement: S10 Fig — A) RT-PCR of total RNA extracted from padi2 morphants show the newly designed MO causes an aberrant splicing resulting to partial retention of intron 6 within the transcript. B) Significant vascular defects in a new padi2 MO compared to standard-injected MO (negative) control siblings (representative figures from 12 injections, repeated 3 times). Note the gaps present in the intersegmental vessels suggesting missing or abnormal formation. (TIF) [file pgen.1006848.s010.tif]

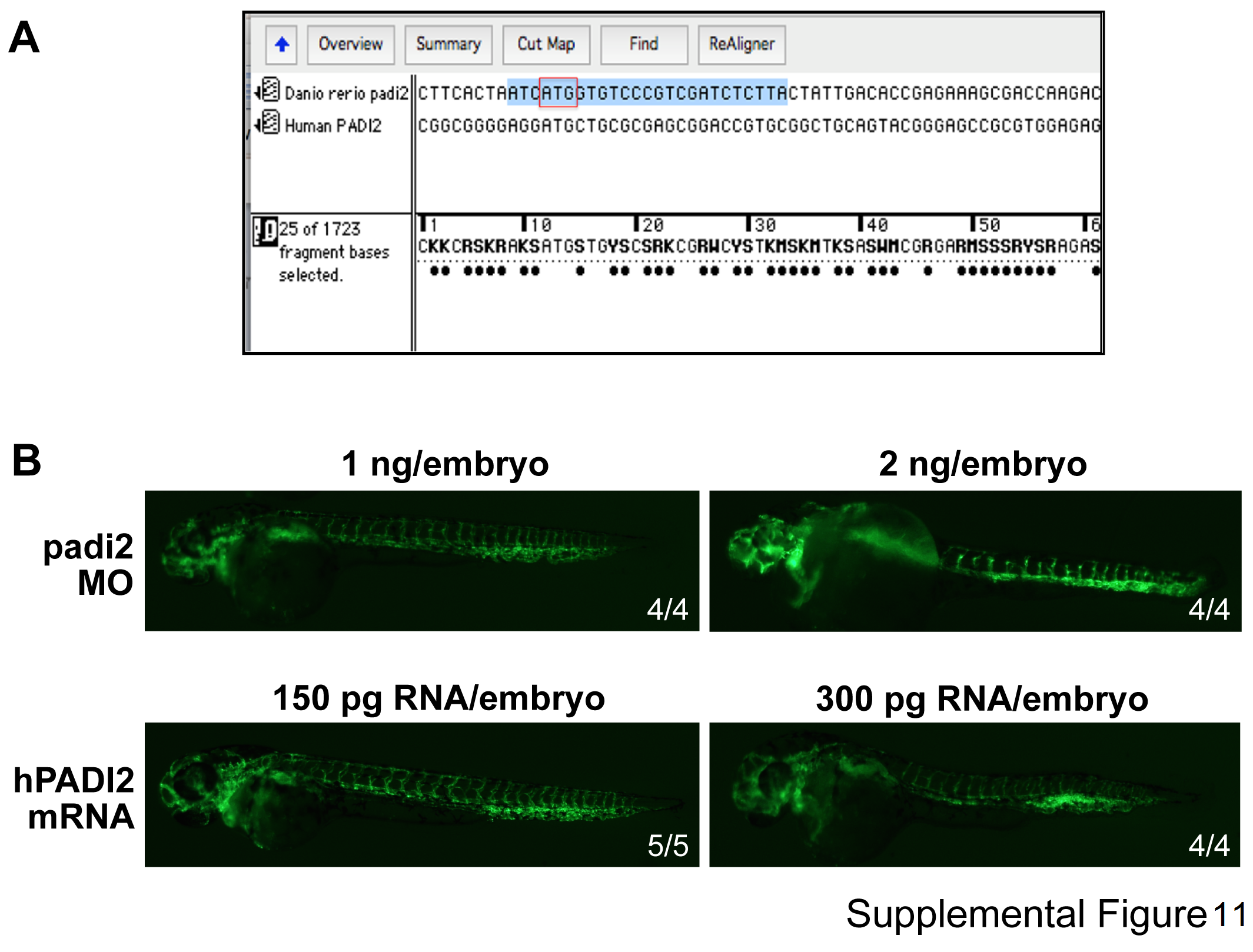

Supplement: S11 Fig — A) Poorly conserved region at the 5’ ATG start site between zebrafish padi2 and human PADI2 mRNA sequence. The designed MO targets the ATG start site (highlighted) of zebrafish padi2. MOs differ by more than five out of 25 nucleotides from their target sequence do not interfere with translation of the targeted mRNA. B) By titrating the dose of human PADI2 (hPADI2) mRNA, we determined that injection of up to 225 pg into early embryos did not cause any phenotypic changes or lethality. Notably, injection of high copy number of hPADI2 mRNA (>250 pg) alone results in significant toxicity in zebrafish. (TIF) [file pgen.1006848.s011.tif]
