# Supplementary material for: Identification of Padi2 as a novel angiogenesis-regulating gene by genome association studies in mice
Source: PLoS Genet. 2017 Jun 15;13(6):e1006848. doi: 10.1371/journal.pgen.1006848 (PMC5491319; doi:10.1371/journal.pgen.1006848)
Supplement: S1 Note — Vessel area ranges from a value of 0.42 mm2 in NZB/BINJ (low angiogenic strain) to 2.05 mm2 in AKR/J (high angiogenic strain). (DOCX) [file pgen.1006848.s012.docx]

**Supplemental Note 1**

|  | |  |  | |  | |
| --- | --- | --- | --- | --- | --- | --- |
|  |  | | |  | |  |
| **Strain** | **NVA** | | | **STD** | |  |
| NZB/BlNJ | 0.415 | | | 0.206 | |  |
| SM/J | 0.692 | | | 0.064 | |  |
| BPN/3J | 0.699 | | | 0.136 | |  |
| I/LnJ | 0.763 | | | 0.153 | |  |
| MA/MyJ | 0.780 | | | 0.050 | |  |
| LG/J | 0.814 | | | 0.187 | |  |
| NZL/LtJ | 0.849 | | | 0.120 | |  |
| NZW/LacJ | 0.865 | | | 0.141 | |  |
| CE/J | 0.865 | | | 0.446 | |  |
| P/J | 0.882 | | | 0.135 | |  |
| NOD/LtJ | 0.925 | | | 0.149 | |  |
| BPL/1J | 0.928 | | | 0.196 | |  |
| C57BR/cdJ | 0.965 | | | 0.128 | |  |
| SEA/GnJ | 0.966 | | | 0.183 | |  |
| DDY/JclSidSeyFrk | 0.981 | | | 0.383 | |  |
| C57BL/6J | 1.001 | | | 0.212 | |  |
| NOR/LtJ | 1.038 | | | 0.174 | |  |
| CBA/J | 1.041 | | | 0.215 | |  |
| FVB/NJ | 1.055 | | | 0.163 | |  |
| C57BL/10J | 1.075 | | | 0.114 | |  |
| SJL/J | 1.109 | | | 0.173 | |  |
| BUB/BnJ | 1.118 | | | 0.169 | |  |
| DBA/1J | 1.121 | | | 0.176 | |  |
| A/J | 1.151 | | | 0.235 | |  |
| RIIIS/J | 1.163 | | | 0.273 | |  |
| NON/LtJ | 1.207 | | | 0.187 | |  |
| KK/HlJ | 1.254 | | | 0.146 | |  |
| TALLYHO/JngJ | 1.260 | | | 0.103 | |  |
| 129X1/SVJ | 1.260 | | | 0.263 | |  |
| Balb/cJ | 1.289 | | | 0.241 | |  |
| LP/J | 1.318 | | | 0.233 | |  |
| MRL/MpJ | 1.319 | | | 0.302 | |  |
| BPH/2J | 1.344 | | | 0.264 | |  |
| DBA/2J | 1.345 | | | 0.273 | |  |
| 129T2/SvEms | 1.453 | | | 0.269 | |  |
| C3H/HeJ | 1.501 | | | 0.179 | |  |
| SWR/J | 1.693 | | | 0.053 | |  |
| C58/J | 1.797 | | | 0.558 | |  |
| Balb/cByJ | 1.924 | | | 0.326 | |  |
| 129S1/SvImJ | 1.943 | | | 0.376 | |  |
| C57BLKS/J | 1.990 | | | 0.278 | |  |
| AKR/J | 2.046 | | | 0.265 | |  |
