# Supplementary material for: Identification of Padi2 as a novel angiogenesis-regulating gene by genome association studies in mice
Source: PLoS Genet. 2017 Jun 15;13(6):e1006848. doi: 10.1371/journal.pgen.1006848 (PMC5491319; doi:10.1371/journal.pgen.1006848)
Supplement: S2 Note — (DOCX) [file pgen.1006848.s013.docx]

**Supplemental Note 2**

#### Padi2: [Mm01341648_m1](https://www.thermofisher.com/order/genome-database/details/ge/Mm01341648_m1?CID=&ICID=)

**Padi3**: Mm00478075_m1

**PADI2**: Hs01042504_m1

#### Slc38a1: [Mm00506391_m1](https://www.thermofisher.com/order/genome-database/details/ge/Mm00506391_m1?CID=&ICID=)

**SLC38A1**: Hs01562168_m1

#### Padi4: [Mm01341658_m1](https://www.thermofisher.com/order/genome-database/details/ge/Mm01341658_m1?CID=&ICID=)

#### PADI4: [Hs01057483_m1](https://www.thermofisher.com/order/genome-database/details/ge/Hs01057483_m1?CID=&ICID=)

#### Irf2bp2: [Mm01239804_g1](https://www.thermofisher.com/order/genome-database/details/ge/Mm01239804_g1?CID=&ICID=)

#### IRF2BP2: Hs00766250_m1

**bActin**: Mm00478075_m1

#### Gapdh: [Mm99999915_g1](https://www.thermofisher.com/order/genome-database/details/ge/Mm99999915_g1?CID=&ICID=)

**bACTIN**: Hs01060665_g1

**18S**: Hs0300363631_g1
