# Supplementary material for: Identification of Padi2 as a novel angiogenesis-regulating gene by genome association studies in mice
Source: PLoS Genet. 2017 Jun 15;13(6):e1006848. doi: 10.1371/journal.pgen.1006848 (PMC5491319; doi:10.1371/journal.pgen.1006848)
Supplement: S3 Note — (DOCX) [file pgen.1006848.s014.docx]

**Supplemental Note 3**

Padi2 (Abcam, Cambridge, MA, USA); 1:1000

Padi4 (LifeSpan Biosciences, Seatle, WA, USA); 1:1000

Citrulline (Abcam, Cambridge, MA, USA); 1:1000

Abcg2 (Abcam, Cambridge, MA, USA); 1:1000

Beta-Actin (Sigma-Aldrich, [St. Louis, MO](https://www.google.com/search?biw=1920&bih=950&q=St.+Louis&stick=H4sIAAAAAAAAAOPgE-LUz9U3sLC0SK5U4gAxzcoryrW0spOt9POL0hPzMqsSSzLz81A4VhmpiSmFpYlFJalFxQDMHhGVQwAAAA&sa=X&ved=0ahUKEwigoMGS7LrSAhWF5iYKHUvvCGgQmxMIowEoATAW), USA); 1:10,000
