# Supplementary material for: Identification of Padi2 as a novel angiogenesis-regulating gene by genome association studies in mice
Source: PLoS Genet. 2017 Jun 15;13(6):e1006848. doi: 10.1371/journal.pgen.1006848 (PMC5491319; doi:10.1371/journal.pgen.1006848)
Supplement: S4 Note — (DOCX) [file pgen.1006848.s015.docx]

**Supplemental Note 4**

padi2-i6e7_e5-e8F1 GTGTTGCGCACTAATGGACC

padi2-i6e7_e5-e8R1 CCTCCACAGGCTTTAGGGTG

padi2-i6e7_e5-e8F7 ATGGTGTTGCGCACTAATGGA

padi2-i6e7_e5-e8R7 TCCACAGGCTTTAGGGTGTTT

padi2-i6e7_e5-e8F10 GACATGTCCAAGATGGTGTTGC

padi2-i6e7_e5-e8R10 ACAGGCTTTAGGGTGTTTGG

PCR Conditions:

95^o^C-2 min, 45 cycles {95^o^C- 30 min, 60^o^C- 30min, 72^o^C- 4min} 72^o^C 5min.
